# NK Cell-Based Immune Checkpoint Inhibition

**DOI:** 10.3389/fimmu.2020.00167

**Published:** 2020-02-13

**Authors:** Muhammad Khan, Sumbal Arooj, Hua Wang

**Affiliations:** ^1^Department of Oncology, The First Affiliated Hospital, Institute for Liver Diseases of Anhui Medical University, Hefei, China; ^2^Department of Biochemistry, University of Sialkot, Sialkot, Pakistan

**Keywords:** natural killer cell (NK), cancer immunotherapy (CI), immune checkpoint inhibitors (ICI), immune checkpoint, immune therapeutics

## Abstract

Immunotherapy, with an increasing number of therapeutic dimensions, is becoming an important mode of treatment for cancer patients. The inhibition of immune checkpoints, which are the source of immune escape for various cancers, is one such immunotherapeutic dimension. It has mainly been aimed at T cells in the past, but NK cells are a newly emerging target. Simultaneously, the number of checkpoints identified has been increasing in recent times. In addition to the classical NK cell receptors KIRs, LIRs, and NKG2A, several other immune checkpoints have also been shown to cause dysfunction of NK cells in various cancers and chronic infections. These checkpoints include the revolutionized CTLA-4, PD-1, and recently identified B7-H3, as well as LAG-3, TIGIT & CD96, TIM-3, and the most recently acknowledged checkpoint-members of the Siglecs family (Siglec-7/9), CD200 and CD47. An interesting dimension of immune checkpoints is their candidacy for dual-checkpoint inhibition, resulting in therapeutic synergism. Furthermore, the combination of immune checkpoint inhibition with other NK cell cytotoxicity restoration strategies could also strengthen its efficacy as an antitumor therapy. Here, we have undertaken a comprehensive review of the literature to date regarding NK cell-based immune checkpoints.

## Introduction

**Figure 1:**
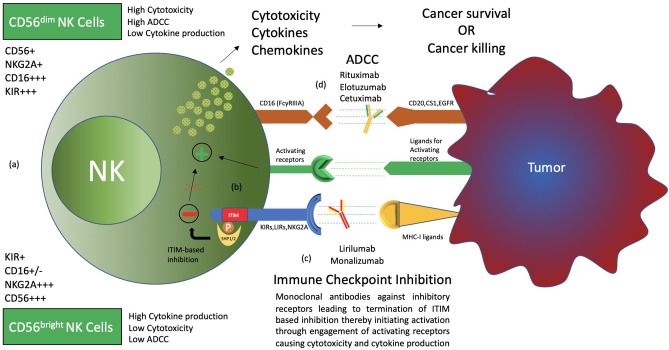
Types and functions of NK cells. **(a)** CD56^bright^ CD16^−^ and CD56^dim^ CD16^+^ NK cells, termed immature and mature NK cells, respectively, are identified to have functional differences. CD56^bright^ NK cells produce more cytokines, while CD56^dim^ CD16^+^ NK cells are more cytotoxic and carry out ADCC (antibody-dependent cell-mediated cytotoxicity). **(b)** NK cell surface receptors, both activating and inhibitory receptors, carry out NK cell functions through a balance of signals. Inhibitory receptors detect MHC-I ligands on normal cells, and if present, activating signals are terminated, thereby maintaining “self-recognition.” These receptors carry ITIM motifs in their cytoplasmic tail, which recruit SHP1/2 through phosphorylation to carry out its function. Such inhibition is termed “dominant inhibition.” **(c)** Inhibitory receptors are exploited by cancer through upregulation of ligands, thereby avoiding destruction by NK cells. Hence, antibodies such as lirilumab and monalizumab are developed to block such interaction and enhance NK cell cytotoxicity toward cancer cells. This phenomenon is termed immune checkpoint inhibition. **(d)** The presence of CD16 receptors on NK cells makes them able to carry out ADCC. Therefore, several antibodies, such as rituximab, elotuzumab, and cetuximab, have been clinically evaluated for synergism with immune checkpoint inhibitors.

Human natural killer cells constitute 15% of all circulating lymphocytes (1). Discovered in the 1970s, NK cells have mainly been associated with the killing of microbially infected and malignantly transformed allogeneic and autologous cells (2–4). NK cells demonstrate antitumor cell cytotoxicity without prior sensitization and production of cytokines as well as chemokines that regulate various immune responses (5). Recent advancements suggest that the actual role of natural killer cells is not restricted to the killing of malignantly transformed and virally infected cells but also plays a circumstantial role in affecting the players of the adaptive immune system such as DCs and T cells, either directly or indirectly, expanding on its functional domain (5–7). Furthermore, NK cell subsets exhibit major functional differences in their cytotoxicity, cytokine production, and homing capabilities (6). Based on CD56 density on the cell surface, NK cells are divided into CD56^bright^ and CD56^dim^, which have different phenotypic properties. CD56^bright^ NK cells have the capacity to produce abundant cytokines, while CD56^dim^ NK cells are more cytotoxic and express more immunoglobulin-like receptors as well as FcγRIII (Fcγ receptor III, also known as CD16) (Figure 1) (1, 6, 8, 9).

Both activating and inhibitory receptors are expressed on the surface of NK cells, contributing to the execution of the functions performed by the NK cell (10–17) (Figure 1). Inhibitory receptors specific for MHC-I (major histocompatibility complex class I) antigens tightly regulate NK cell-mediated cytotoxicity and lymphokine production. The inhibitory signal from the MHC-I specific receptor is essential for hematopoietic target cells to avoid destruction by NK cells. This concept is termed the “missing self” and was originally proposed by Ljunggren and Karre (17–20). Such MHC-I-recognizing inhibitory receptors form three families of NK-cell surface receptors, namely KIRs (killer cell immunoglobulin-like receptors), LIRs (Leukocyte immunoglobin-like receptors), and NKG2A (natural killer group 2 A) (10–14, 21). KIRs, members of the immunoglobulin superfamily, are type I transmembrane molecules that recognize classical human leukocyte antigens A, B, and C (HLA class Ia) (10, 11, 13). LIRs, also known as ILTs (immunoglobin-like transcripts), form the second set of receptors and mainly recognize non-classical HLA-G (class Ib) molecules, in addition to HLA class Ia. LIRs belong to the same Ig superfamily as KIRs. NKG2A, a member of the NKG2 group of seven receptors, namely A, B, C, D, E, F, and H, dimerizes with CD94 to form the NKG2A/CD94 receptor. It belongs to the C-type lectin family of receptors that recognizes non-classical HLA-E class I molecule as its ligand (22).

Destruction by NK cells not only requires the detection of MHC-I molecules on transforming cells by inhibitory receptors but also the activation of the NK cell by activating receptors (10–17). Natural cytotoxicity receptors (NCRs) represent the group of natural killer cell surface activating receptors that includes NKp46, NKp30, and NKp44. These receptors, as well as NKG2D and DNAM-1 (DNAX accessory molecule-1), recognize ligands expressed on the surface of virally infected or malignantly transformed cells (15, 23, 24). A number of coreceptors (2B4, NKp80, NTB-A, and CD59) are also expressed, which function only in combination with other activating receptors (23, 25). CD16 (or FcγRIII), also an activating receptor, is expressed mainly by the CD56^dim^ NK-cell subset and is essential for antibody-dependent cellular cytotoxicity (ADCC) against IgG-coated target cells (1, 26) (Figure 1).

**Figure 2:**
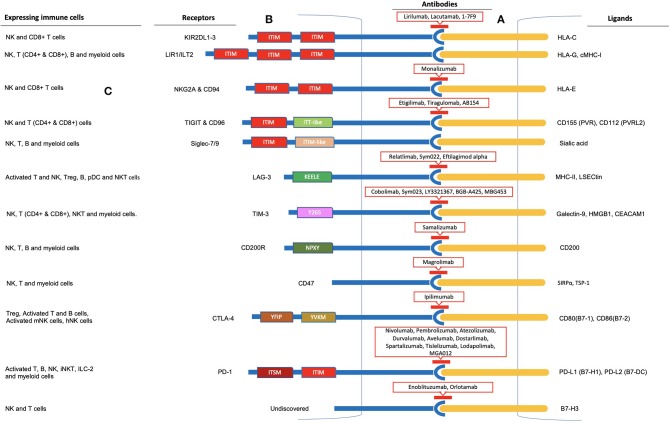
Immune checkpoint inhibition observed in Natural Killer cells. **(A)** Inhibitory receptor-ligand interaction leading to immune escape of cancer cells is termed immune checkpoint inhibition. Inhibitory receptors expressed on the surface of NK cells are illustrated as blue rods, and ligands for these receptors expressed by tumor cells are illustrated as orange rods. **(B)** Rectangular boxes represent the intracellular domains of the receptors through which inhibition is carried out. Several of these receptors (KIR, ILT2, NKG2A & CD94, TIGIT & CD96, Siglec-7/9, PD-1, and SIRPα) bear 1-3 ITIMs in their cytoplasmic tail and observe ITIM-based inhibition. In addition, TIGIT and PD-1 cytoplasmic tails contain an ITT-like and ITSM motif, respectively. LAG-3, TIM-3, CD200, and CTLA-4 lack an ITIM motif in their cytoplasmic tails. Instead, they have special intracellular tyrosine motifs such as KEELE, Y265, NPXY, and YVKM and YFIP, respectively, which are implicated in carrying out the inhibition process. Antibodies to these receptors are shown within red-outlined boxes. **(C)** Moreover, several other immune cells, including T cells, B cells, and myeloid cells, express these receptors on their surfaces, as shown on the left panel for each immune checkpoint receptor.

Tumors evade the immune system through the establishment of an immune-suppressive tumor microenvironment (27). Immune evasion involving NK cells encompasses several mechanisms (5, 28–30). Hypoxia and the release of various cytokines and soluble factors by tumor cells or other components of the tumor microenvironment such as transforming growth factor-β (TGFβ), IL-6, IL-10, tryptophan catabolites, prostaglandin E_2_ (PGE_2_), dickkopf-related protein 2 (DKK2), idoleamine 2,3-dioxygenase (IDO), soluble HLA-G, soluble NKG2D ligands, and galactin-3 (soluble inhibitory receptor for NKp30) have been reported to decrease the activation of NK cells and their cytotoxic activity, production of IFNγ, as well as expression and activation of its activating receptors (28–36). Furthermore, ligand shedding for activating receptors and upregulation of ligands for inhibitory receptors by tumor cells have also been revealed (25, 37). Hence, various strategies have been developed to restore NK cell functions, including adoptive cell transfer, cytokine therapies, and monoclonal antibodies targeting activating as well as inhibitory receptors and the tumor microenvironment (5, 25, 38–44). The exploitation of the NK cell inhibitory receptors by tumors for immune evasion is one such mechanism, termed immune checkpoint inhibition, and has been proven to be the most effective and appreciated therapeutic target (Box 1). Several such inhibitory immune checkpoints involving NK cells other than the MHC class I-associated inhibitory receptors have been identified, including the classical CTLA-4 and PD-1 receptors and TIGIT & CD96, LAG-3, and TIM-3 (Figure 2). B7-CD28 family members such as B7-H3, VISTA (PD-1H), and B7-H7 have recently been reported as plausible targets for such inhibition in regard to NK cells. More recently identified as NK cell-based immune checkpoint inhibition targets are the Siglec family receptors, namely Siglec-7 and -9, CD200, and CD47. Here, we review the recent advances and updates in NK cell-based immune checkpoint inhibition.

Box 1 **Immune checkpoint inhibition**

The immune system consists of innate and adaptive immune components that, upon activation, eliminate infectious agents and cancer cells. Inhibitory pathways exist that normally maintain self-tolerance and counter-balance the activation process in order to avoid excessive damage and limit collateral tissue damage during the anti-microbial or anti-cancer immune response. These inhibitory pathways, consisting of receptors and ligands, are termed “immune checkpoints” and are utilized by the cancer cells to evade immune destruction. Inhibition of these checkpoints by developing monoclonal antibodies, thereby relieving the immune cells from inhibition and enabling them to recognize the cancer cells in order to eliminate them, is termed as “immune checkpoint inhibition,” and the agents are called “immune checkpoint inhibitors” (45). Ipilimumab, a CTLA-4 blocker, became the first approved immune checkpoint blocking anti-cancer drug in 2011 in the United States (46). **James P. Allison** and **Tasuku Honjo** were honored with the **Nobel Prize in Physiology or Medicine** in 2018 for their work and discoveries in basic science that allowed the development of checkpoint inhibitor therapies (47).

## MHC-I-Specific NK Inhibitors

NK cells avoid the killing of healthy autologous cells through MHC-I specific inhibitory receptors such as KIRs and NKG2A & CD94. Ligation results in a transient abortion of activation signals rather than anergy or apoptosis of natural killer cells (18) (Figure 1). These inhibitory receptors carry an inhibitory motif in their cytoplasmic domain called the immunoreceptor tyrosine-based inhibition motif (ITIM). Tyrosine phosphatases SHP-1/SHP-2 are recruited by ITIMs to carry out their function (48). Inhibitory receptors carrying such an inhibitory motif in their cytoplasmic tail constitute a specific group of receptors with various functions. Such inhibition is termed “dominant inhibition,” as it inhibits signaling at a proximal level (Box 2). Other NK cells surface inhibitory receptors, such as TIGIT, PD-1, and the Siglecs family inhibitory receptors, also possess ITIM containing cytoplasmic tails, while some of the inhibitory receptors lack this cytoplasmic inhibitory motif (Box 2).

Box 2 **ITIM-based inhibition and intracellular signaling in NK cells**

**Dominant inhibition** 

Negative regulation exhibited by classical NK-cell inhibitory receptors is distinct from negative feedback, as it prevents the activation from happening by blocking activation signals at an early stage (16, 18, 19). A transient abortion of activation signals is achieved rather than anergy or apoptosis as a result of this negative regulation. This type of inhibitory regulation is called dominant inhibition, as the downstream activation signals are prevented from occurring at all (16).

**ITIM-based inhibition** 

This dominant inhibition by NK-cell inhibitory receptors is carried out through a cytoplasmic immunoreceptor tyrosine-based inhibition motif (ITIM). Such ITIM-bearing receptors are members of several receptor families and are expressed in many cell types, such as leukocytes, mast cells, monocytes, dendritic cells, and macrophages with various cellular functions that include allergy, autoimmunity, graft vs. host disease, phagocytosis of red blood cells, and neuronal plasticity in the brain (18, 49). The ITIM domain, at least one, is present in over 300 integral membrane proteins, both type I and type II, as revealed in bioinformatics analyses of entire genes of the human genome (49). Inositol (SHIP1 and SHIP2) or tyrosine (SHP1 and SHP2) phosphatases that contain Src homology (SH) 2 domain are recruited by ITIM upon receptor-ligand binding. Nonetheless, SHP1 and SHP2 tyrosine phosphatases represent the majority ITIM-bearing receptor binders (16, 50). Functional characterization of inhibitory KIR revealed the precise ITIM sequence as the binding site for SHP1 to be “V/IxYxxL/V” (“x” indicates a non-conserved position, “/” indicates either). A sequence of 25 amino acids separates the two ITIMs in the cytoplasmic tail of the single inhibitory receptor, thereby providing a binding site for the tandem SH2 domains of SHP1 and SHP2 (48, 50).

SHP1 recruitment, leading to Vav1 dephosphorylation and Crk phosphorylation by c-Abl, have been the intracellular signaling pathways involved in the inhibition process observed in NKG2A/CD94 and KIR. KIRs recruit SHP1 and SHP2 for its inhibitory action but not SHIP (16, 18, 19, 51, 52). On the other hand, LIR (leukocyte immunoglobulin-like receptors LIR1-5) cytoplasmic tails contain two to four ITIMs. ILT2, the one mainly involved in NK cell inhibition, contains three ITIMs in its cytoplasmic tail (53). The TIGIT cytoplastic tail contains one immunoreceptor tail tyrosine (ITT)-like motif in addition to ITIM. Grb2 and β-arrestin2 phosphorylation by Fyn and Lck leads to the recruitment of SHIP1 to downregulate PI3K, MAPK, and NF-κB (nuclear factor-κB) signaling, resulting in a robust reduction of NK cell-mediated cytotoxicity, its granule polarization and production of cytokines (54–57). Similar to classical NK-cell inhibitory receptors NKG2A/CD94 and KIRs, inhibitory Siglecs also contain one or more ITIM and ITIM-like motifs in their cytoplasmic tail. Upon ligation, ITIMs are phosphorylated by Src family kinases recruiting and activating SRC homology 2 (SH2)-domain-containing proteins, mainly the tyrosine phosphatases SHP1 and SHP2 or the SOCS3 (suppressor of cytokine signaling 3) protein (58). PD-1 carries one immunoreceptor tyrosine-based switch motif (ITSM) in addition to one ITIM in its cytoplasmic tail. ITSM is required for the inhibitory functions associated with PD-1 triggering, such as PI3K/Akt activation and suppression of interleukine-2 production, upon phosphorylation by SHP1 or SHP2. Particularly, Y248 (PD-1 ITSM) was found to interact with SHP1 and SHP2, and its association with SHP2 is required for the inhibition of PI3K/Akt activation (59–61). SIRPα has three extracellular Ig domains and an intracellular tail with two immuno-receptor tyrosine-based inhibitory motifs (ITIMs) (62). SIRPα-contained ITIM motifs inhibit phagocytosis and cellular attachment through SHP1 upon interaction with CD47, and members of the JAK/STAT family signaling pathway have been identified as potential downstream mediators of SIRPα signaling (63, 64).

**ITIM-lacking inhibition** 

CTLA-4 lacks the classic signaling motif (ITIM) in its cytoplasmic tail, and phosphorylation of CTLA-4 tyrosines (YVKM and YFIP) fails to allow for single or tandem SHP2 SH2 domain binding (65). It directly inhibits the activation of Akt, but not of PI3K, by association with protein phosphatase 2A (PPA2) (59–61, 66). However, a CTLA-4 mutant lacking PP2A-binding sites appeared to show increased inhibitory function. Walker et al. termed CTLA-4 intracellular signaling confusing and unclear due to the contradictory nature of the medical literature and proposed to consider moving away from the signaling view toward a quantitative conception of ligand competition (67). LAG-3 has a unique cytoplasmic tail containing three regions. The first region contains a serine phosphorylation site, while the KIEELE motif that is contained in the second region has been revealed to be essential for the inhibitory function of Lag-3 in effector CD4^+^ T cells. The third region contains glutamic acid-proline (EP) repeats. These regions are conserved in humans and mice (59, 68). The TIM-3 cytoplasmic tail contains five conserved tyrosine residues, with two tyrosines (residues 256 and 263 in mouse TIM-3) being particularly important (69). In the absence of ligand binding, TIM-3 is associated with HLA-B-associated transcript 3 (Bat3), which protects the cell from TIM-3-mediated inhibition. Ligand binding (Galec-9 and CEACAM-1) leads to the phosphorylation of tyrosine residues (Y256 and Y263 in mouse, and Y265, a corresponding tyrosine residue to Y256, in human) causing disruption of the association between TIM-3 and Bat-3 and thereby allowing TIM-3-mediated inhibition to occur (59, 70). TIM-3 can associate with interleukin-inducible T-cell kinase (ITK), the Src kinases Fyn and Lck, and the p85 phosphatidylinositol 3-kinase (PI3K) adaptor protein to positively or negatively regulate IL-2 production via NF-κB/NFAT signaling pathways (69–71). CD200R also lacks the classical ITIM in its cytoplasmic tail but contains three tyrosine residues (Y286, Y289, and Y297), one of which (Y297) is located in an NPxY motif. Phosphorylation of tyrosine residues (majorly, Y286 and Y297) leads to the recruitment of inhibitory adaptor proteins Dok1 and Dok2 and the subsequent inhibition of Ras/mitogen-activated protein kinase (RasGAP) activation (72, 73).

## KIRs

The KIR family (also known as CD158) is a diversified and polymorphic group of NK cell receptors comprising inhibitory and activating KIRs, each recognizing a specific HLA class I allotype (HLA-A, -B, or -C) as ligand (13). The inhibitory KIR2DL1, KIR2DL2, and KIR2DL3 recognize HLA-C as their ligand while HLA-B and HLA-A serve as ligands to other KIRs, including inhibitory KIR3DL1 and KIR3DL2. In addition to NK cells, T cell subsets and iNKT cells (invariant natural killer T cells) also expressed KIR (74). MHC-I molecules (HLA-A, -B, and -C), ligands for KIRs and KIRs, themselves exhibit extensive natural polymorphism (75). The diverse allelic combination of the KIR genes (a total of 17 KIR genes on chromosome 19q13, 14), the polymorphism within each gene, and each NK cell expressing some of the KIRs make this complex KIR repertoire capable of recognizing minute alterations in MHC-I expression (51, 76–81).

**Figure 3:**
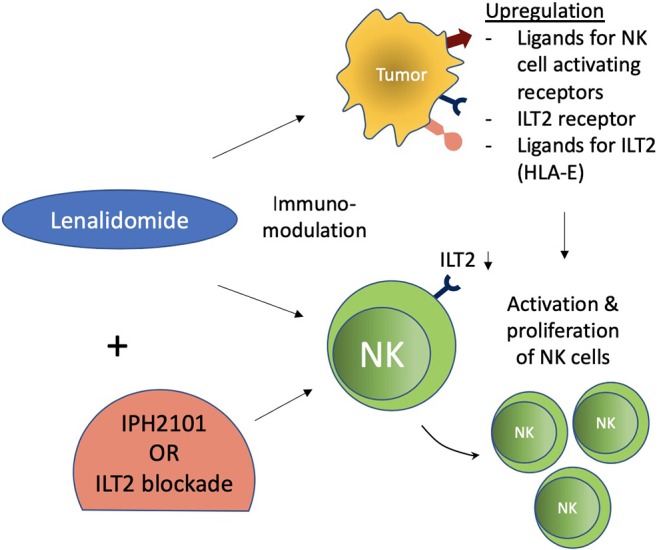
Immunomodulatory effect of lenalidomide on NK cells. Lenalidomide upregulates ligands for NK cell-activating receptors in multiple myeloma and augments NK cell function. Lenalidomide also increases ILT2 expression on CLL and decreases it on NK cells, while its ligand (HLA-E) is reestablished on leukemic cells. These immunomodulatory effects of NK cells were associated with increased NK cell activation and proliferation. ILT2 blockade in this setting further potentiated NK cell functions.

IPH2101 and lirilumab (IPH2102/BMS-986015) are IgG4 monoclonal antibodies (mAbs) targeting KIR2DL1/2/3 NK inhibitory receptors that are currently in clinical evaluation and development as monotherapy or in combination with other agents, including a molecular targeted agent (lenalidomide), monoclonal antibodies (elotuzumab & rituximab), and immune checkpoint blockers (ipilimumab & nivolumab) (82–99) (Table 1). IPH2101 is clinically evaluated in various hematologic (AML, CLL, NHL) or solid malignancies (breast and ovarian cancers). A dose of up to 10 mg/kg of IPH2101 was reported to be well-tolerated (82–85). So far, IPH2101 as monotherapy has failed to impress clinically in patients with multiple myeloma (MM). A dose-escalation, phase I trial of IPH2101 in MM (relapsed/refractory) as monotherapy, with the primary objective of assessing the maximum dose tolerable and limiting toxicity, reported acceptable safety and tolerability without any evidence of autoimmunity. However, just 11 (34%) patients achieved the best response of stable disease, as evaluated by the International Myeloma Working Group (IMWG) criteria (82). It was revealed that infusion of IPH2101 in MM patients had reduced NK cell surface expression of the KIR2D receptor and the responsiveness of the NK cell (86). Preclinical evidence has demonstrated functional augmentation of NK cells and ligand upregulation for activation of NK cell surface receptors on MM cells by lenalidomide, which, in combination with IPH2101, have shown potent *in vivo* rejection of lenalidomide-resistant tumor (87) (Figure 3). IPH2101 and lenalidomide as “dual immunotherapy” for MM patients has been reported to achieve a median progression-free survival of 24 months, five objective responses with acceptable toxicity (five severe AEs), and no autoimmunity. Overall, this combination holds promise and warrants further clinical evaluation in MM patients despite the failure of IPH2101 as a single agent (88, 89). A phase II trial of lirilumab was terminated because of failure to meet the objective response criteria (50% decline in M-protein) set for MM patients, with only one (11%) and six (66%) of a total of nine patients enrolled achieving minimal response and stable disease (90). However, *in vitro* elotuzumab-mediated cell-killing was enhanced by lirilumab and showed synergism in potentiating anti-tumor efficacy in KIR2DL3-transgenic and RAG-deficient mice (91). *In vitro* augmentation of elotuzumab-mediated ADCC and *in vivo* synergism in mediating potent elotuzumab anti-MM activity by lirilumab were also reported by Sola et al., setting the rationale for clinical evaluation of this combination in MM patients (92). A phase I (NCT2252263) study evaluating elotuzumab and lirilumab in combination in multiple myeloma patients is currently in development.

**Table 1:** Clinical trials evaluating the safety, tolerability and efficacy of NK cell-based immune checkpoint inhibitors or potential immune checkpoint inhibitors for NK cell-based immunotherapy.

**Receptor**	**National clinical trial no**.	**Phase, allocation**	**Sponsors**	**Participants**	**Drugs**	**Combinations**	**Status**	**Disease**	**First posting date**
KIR	NCT00552396	I, Non-randomized	Innate Pharma	32	Anti-KIR (1-7F9)	Single agent	Completed	Multiple myeloma	November 1, 2007
	NCT00999830	II, Randomized	Innate Pharma	27	IPH2101	Single agent	Completed	Patients with multiple myeloma in stable partial response after a first line therapy	October 22, 2009
	NCT01256073	I, Non-randomized	Innate Pharma	21	IPH2101	Single agent	Completed	Acute myeloid leukemia	December 8, 2010
	NCT01222286	II, Randomized	Innate Pharma	30	IPH2101	Single agent	Completed	Smoldering multiple myeloma	October 18, 2010
	NCT01217203	I, Non-randomized	Innate Pharma	15	IPH2101, lenalidomide	IPH2101 plus lenalidomide	Completed	Patients with multiple myeloma experiencing a first or second relapse	October 8, 2010
	NCT01687387	II, Randomized	Innate Pharma	152	Lirilumab (IPH2102), placebo	Lirilumab vs. placebo	Completed	Elderly Patients with Acute Myeloid Leukemia (AML) in First Complete Remission	September 18, 2012
	NCT01750580	I, Non-randomized	Bristol-Myers Squibb	22	Lirilumab, ipilimumab	Lirilumab plus ipilimumab	Completed	Selected advanced tumor	December 17, 2012
	NCT02252263	I, Randomized	Innate Pharma	44	Elotuzumab, lirilumab (BMS-986015), urelumab	Elotuzumab plus lirilumab or urelumab	Completed	Multiple myeloma	September 30, 2014
	NCT02481297	II, Non-randomized	M.D. Anderson Cancer Center, Bristol-Myers Squibb	8	Lirilumab, rituximab	Lirilumab plus rituximab	Active	Leukemia, chronic lymphocytic leukemia, lymphocytic leukemia	June 25, 2015
	NCT03203876	I, Non-randomized	Bristol-Myers Squibb/Ono Pharmaceutical Co., Ltd.	21	Lirilumab, nivolumab, ipilimumab	Lirilumab plus nivolumab or lirilumab plus nivolumab and ipilimumab	Active, not recruiting	Advanced and/or metastatic solid tumors	June 29, 2017
	NCT01714739	I/II, Randomized	Bristol-Myers Squibb	337	Lirilumab, nivolumab, ipilimumab	Lirilumab plus nivolumab or lirilumab plus nivolumab and ipilimumab	Active, not recruiting	Advanced refractory solid tumors	October 26, 2012
	NCT02593045	I, Non-randomized	Innate Pharma	60	IPH4102, a humanized anti-KIR3DL2 monoclonal antibody	Single agent	Active, not recruiting	Relapsed/refractory cutaneous T-cell lymphomas (CTCL)	October 30, 2015
	NCT03902184	II, Non-randomized	Innate Pharma	250	IPH4102, gemcitabine + oxaliplatin	IPH4102 alone or in combination with chemotherapy	Recruiting	Lymphoma, T-Cell lymphoma, T-Cell, cutaneous lymphoma, T-cell, peripheral mycosis fungoides/sezary syndrome	April 3, 2019
NKG2A	NCT02331875	I/II, Non-randomized	Innate Pharma	3	Monalizumab (IPH2201)	Single agent	Terminated (lack of recruitment)	Squamous Cell carcinoma of the oral cavity	January 6, 2015
	NCT02459301	I, Non-randomized	Canadian Cancer Trials Group, Innate Pharma	59	Monalizumab	Single agent	Completed (November 13, 2019)	Gynecologic malignancies	June 2, 2015
	NCT02921685	I, Non-randomized	Institut Paoli-Calmettes, Innate Pharma	18	Monalizumab	Single agent	Recruiting	Hematologic malignancies	October 3, 2016
	NCT02557516	I/II, Non-randomized	Innate Pharma	22	Monalizumab, ibrutinib	Monalizumab plus ibrutinib	Active, not recruiting	Relapsed, refractory or previously untreated chronic lymphocytic leukemia	September 23, 2015
	NCT02643550	I/II, Non-randomized	Innate Pharma, Astrazeneca	140	Monalizumab, cetuximab, anti-PD(L)1	Monalizumab plus cetuximab or monalizumab plus cetuximab plus Anti-PD(L)1	Recruiting	Human Papillomavirus (HPV) (+) and HPV (-) Recurrent or Metastatic HNSCC	December 31, 2015
	NCT02671435	I/II, Non-randomized	MedImmune LLC	501	Durvalumab (MEDI4736), monalizumab	Durvalumab plus monalizumab	Recruiting	Advanced solid tumors	February 2, 2016
	NCT03088059	II, Non-randomized	European Organization for Research and Treatment of Cancer—EORTC	340	Monalizumab, durvalumab, afatinib, palbociclib, standard of care, niraparib, rogaratinib (BAY1163877)	Monalizumab alone or monalizumab plus durvalumab or SOC	Recruiting	Recurrent/metastatic HNSCC	March 23, 2017
	NCT03822351	II, Randomized	MedImmune LLC	300	Monalizumab, durvalumab (MEDI-4736), oleclumab (MEDI-9447) (anti-CD73 antibody),	Durvalumab plus monalizumab or durvalumab plus oleclumab or durvalumab alone	Recruiting	Locally advanced, unresectable (Stage III) non-small cell lung cancer (COAST)	January 30, 2019
	NCT03833440	II, Randomized	Assistance Publique Hopitaux De Marseille	120	Monalizumab, durvalumab, oleclumab, AZD6738 (ATR inhibitor), DOCETAXEL	Durvalumab plus monalizumab or durvalumab plus oleclumab or durvalumab plus AZD6738 or DOCETAXEL alone	Not yet recruiting	Advanced non-small cell lung cancer patients with PD-1 ICI resistance	February 7, 2019
TIGIT	NCT02794571	I, Non-randomized	Genentech, Inc.	300	Tiragolumab (MTIG7192A), atezolizumab	Tiragolumab alone or tiragolumab plus atezolizumab	Recruiting	Advanced or metastatic tumors	June 9, 2016
	NCT03119428	I, Non-randomized	OncoMed Pharmaceuticals, Inc.	33	Etigilimab (OMP-313M32), nivolumab	Etigilimab alone or etigilimab plus nivolumab	Terminated (sponsor decision)	Locally advanced or metastatic solid tumors	April 18, 2017
	NCT03563716	II, Randomized	Genentech, Inc.	135	Tiragolumab (MTIG7192A), atezolizumab	Atezolizumab plus tiragolumab or atezolizumab plus placebo	Active, not recruiting	Locally advanced or metastatic non-small cell lung cancer	June 20, 2018
	NCT03628677	I, Non-randomized	Arcus Biosciences, Inc.	242	AB154 (anti-TIGIT), AB122 (anti-PD-1)	AB154 alone or AB154 plus AB122	Recruiting	Advanced solid malignancies	August 14, 2018
LAG-3	NCT03489369	I	Symphogen A/S	30	Sym022 (anti-LAG-3)	Single agent	Recruiting	Advanced solid tumor malignancies or lymphomas	April 5, 2018
	NCT03311412	I, Non-randomized	Symphogen A/S	102	Sym021 (anti-PD-1), Sym022 (anti-LAG-3), Sym023 (anti-TIM-3)	Sym021 or Sym021 plus Sym022 or Sym021 plus Sym023	Recruiting	Advanced solid tumor malignancies or lymphomas	October 17, 2017
	NCT01968109	I/II, Randomized	Bristol-Myers Squibb	2000	Relatlimab, nivolumab, BMS-986213	Relatlimab alone or relatlimab plus nivolumab	Recruiting	Advanced solid tumors	October 23, 2013
	NCT02061761	I/II, Non-randomized	Bristol-Myers Squibb	109	BMS-986016 (relatlimab) (anti-Lag-3), nivolumab	Relatlimab alone or relatlimab plus nivolumab	Active, not recruiting	Relapsed or refractory hematologic malignancies	February 13, 2014
	NCT02658981	I, Non-randomized	Sidney Kimmel Comprehensive Cancer Center at Johns Hopkins, National Cancer Institute (NCI), Bristol-Myers Squibb	100	Relatlimab, nivolumab, urelumab	Relatlimab plus nivolumab or urelumab plus nivolumab	Recruiting	Glioblastoma, gliosarcoma, recurrent brain neoplasm	January 20, 2016
	NCT02966548	I, Non-randomized	Bristol-Myers Squibb, Ono Pharmaceutical Co. Ltd	45	Relatlimab, nivolumab	Relatlimab alone or relatlimab plus nivolumab	Recruiting	Advanced solid tumors	November 17, 2016
	NCT03044613	I, Non-randomized	Sidney Kimmel Comprehensive Cancer Center at Johns Hopkins, Bristol-Myers Squibb	25	Nivolumab, relatlimab, carboplatin, paclitaxel, radiation	Nivolumab or nivolumab/relatlimab prior to concurrent chemoradiation plus nivolumab or nivolumab/relatlimab	Active, not recruiting	Gastric cancer, esophageal cancer, gastroesophageal cancer	February 7, 2017
	NCT03459222	I/II, Non-randomized	Bristol-Myers Squibb	230	Relatlimab, nivolumab, BMS-986205 (IDO1 inhibitor), ipilimumab	Relatlimab plus nivolumab plus IDO1 inhibitor or relatlimab plus nivolumab plus ipilimumab	Recruiting	Advanced cancer	March 8, 2018
	NCT03493932	I	National Institute of Neurological Disorders and Stroke (NINDS)	20	Nivolumab, relatlimab	Nivolumab plus relatlimab	Recruiting	Glioblastoma	April 11, 2018
	NCT03623854	II	Jonsson Comprehensive Cancer Center, National Cancer Institute (NCI)	20	Nivolumab, relatlimab	Nivolumab plus relatlimab	Recruiting	Advanced Chordoma	August 9, 2018
	NCT03743766	II, Randomized	John Kirkwood Bristol-Myers Squibb	42	Relatlimab, nivolumab	Nivolumab or relatlimab or nivolumab plus relatlimab	Recruiting	Metastatic melanoma	November 16, 2018
	NCT02614833	II, randomized	Immutep S.A.	241	Eftilagimod alpha, paclitaxel	Eftilagimod Alpha plus Paclitaxel or placebo plus paclitaxel	Active, not recruiting	Metastatic breast carcinoma	November 25, 2015
	NCT02676869	I	Immutep Australia Pty. Ltd.	24	IMP321 (eftilagimod alpha), pembrolizumab	IMP321 (Eftilagimod alpha) adjuvant to Anti-PD-1	Active, not recruiting	Unresectable or metastatic melanoma	February 8, 2016
	NCT03252938	I, Non-randomized	IKF Klinische Krebsforschung GmbH at Krankenhaus Nordwest	50	IMP321 (LAG-3Ig fusion protein), avelumab	IMP321 alone or IMP321 plus avelumab	Recruiting	Solid tumor peritoneal carcinomatosis	August 17, 2017
	NCT03625323	II, Non-randomized	Immutep S.A., Merck Sharp & Dohme Corp.	109	Eftilagimod alpha, pembrolizumab	Eftilagimod alpha plus pembrolizumab	Recruiting	NSCLC HNSCC	August 10, 2018
TIM-3	NCT03489343	I, Non-randomized	Symphogen A/S	24	Sym023 (anti-TIM-3)	Single agent	Active, not recruiting	Advanced solid tumor malignancies or lymphomas	April 5, 2018
	NCT02817633	I, Non-randomized	Tesaro, Inc.	873	Cobolimab (TSR-022, an anti-TIM-3 antibody), TSR-042 (dostarlimab, an anti-PD-1 antibody), TSR-033 (an anti-LAG-3 antibody)	Cobolimab alone or cobolimab plus dostarlimab or cobolimab plus dostarlimab plus TSR-033	Recruiting	Advanced solid tumors	June 29, 2016
	NCT03680508	II, Non-randomized	University of Hawaii, Tesaro, Inc.	42	Cobolimab, dostarlimab	Cobolimab plus dostarlimab	Recruiting	Liver cancer	September 21, 2018
	NCT04139902	II, Randomized	Diwakar Davar, Tesaro, Inc.	56	Dostarlimab, cobolimab	Dostarlimab alone or cobolimab plus dostarlimab	Not yet recruiting	Resectable stage III or oligometastatic stage IV melanoma	October 25, 2019
	NCT03099109	I, Non-randomized	Eli Lilly and Company	196	LY3321367(an anti-TIM-3 antibody), Lodapolimab (LY3300054, an anti-PD-L1 antibody)	LY3321367 Alone or LY3321367 plus lodapolimab	Recruiting	Advanced relapsed/refractory solid tumors	April 4, 2017
	NCT03744468	I/II, Non-randomized	BeiGene	162	BGB-A425 (an anti-TIM-3 antibody), tislelizumab (BGB-A317, an anti-PD-1 antibody)	BGB-A425 plus tislelizumab	Recruiting	Advanced solid tumors	November 16, 2018
	NCT02608268	I/II, Non-randomized	Novartis Pharmaceuticals	250	Spartalizumab, MBG453 (an anti-TIM-3 antibody)	MBG453 alone or MBG453 plus Spartalizumab	Recruiting	Advanced malignancies.	November 18, 2015
	NCT03066648	I, Randomized	Novartis Pharmaceuticals	235	MBG453 (an anti-TIM-3 antibody), decitabine, spartalizumab,	MBG453 alone or MBG453 plus decitabine or MBG453 plus spartalizumab or MBG453 plus decitabine plus spartalizumab	Recruiting	AML or high risk myelodysplastic syndrome	February 28, 2017
	NCT03961971	I, Non-randomized	Sidney Kimmel Comprehensive Cancer Center at Johns Hopkins, Novartis Pharmaceuticals	15	MBG453 (an anti-TIM-3 antibody), spartalizumab (PDR001)	MBG453 plus spartalizumab	not yet recruiting	Recurrent GBM	May 23, 2019
CD200	NCT00648739	I/II, Non-randomized	Alexion Pharmaceuticals	26	Samalizumab (ALXN6000)	Single agent	Terminated	Relapsing or refractory CLL or MM	April 1, 2008
	NCT02987504	I, Non-randomized	Alexion Pharmaceuticals, Quintiles, Inc.	10	Samalizumab (ALXN6000)	Single agent	Terminated	Advanced solid tumors	December 9, 2016
CD47	NCT02953509	I/II, Non-randomized	Forty Seven, Inc., The Leukemia and Lymphoma Society	72	Magrolimab (Hu5F9-G4), rituximab	Magrolimab plus rituximab	Recruiting	Relapsed/refractory B-cell non-hodgkin's lymphoma	November 2, 2016
	NCT03248479	I, Non-randomized	Forty Seven, Inc., California Institute for Regenerative Medicine (CIRM)	96	Magrolimab, azacitidine	Magrolimab alone or magrolimab plus azacitidine	Recruiting	Acute myeloid leukemia myelodysplastic syndromes	August 14, 2017
	NCT02953782	I/II, non-randomized	Forty Seven Inc., California Institute for Regenerative Medicine (CIRM)	112	Magrolimab, cetuximab	Magrolimab plus cetuximab	Recruiting	Solid tumor and advanced colorectal cancer	November 3, 2016
	NCT03527147	I, Non-randomized	Acerta Pharma BV, AstraZeneca	88	Magrolimab, rituximab, acalabrutinib, AZD9150, AZD6738, AZD5153	Magrolimab or rituximab plus acalabrutinib	Recruiting	Relapsed or refractory aggressive non-hodgkin's lymphoma	May 17, 2018
	NCT03869190	I/II, Randomized	Hoffmann-La Roche, Forty Seven Inc., Tesaro Inc., Seattle Genetics and Astellas, Sanofi	305	Magrolimab, atezolizumab, enfortumab vedotin, niraparib, isatuximab, linagliptin, tocilizumab	Atezolizumab plus magrolimab	Recruiting	Urothelial carcinoma	March 11, 2019
	NCT03922477	I, Non-randomized	Hoffmann-La Roche	21	Magrolimab, atezolizumab (anti-PD-L1 antibody)	Magrolimab plus atezolizumab	Recruiting	Acute myeloid leukemia	April 22, 2019
B7-H3	NCT02923180	II, Non-randomized	Sidney Kimmel Comprehensive Cancer Center at Johns Hopkins, MacroGenics	33	Enoblituzumab (MGA271)	Neoadjuvant enoblituzumab	Active, not recruiting	Localized intermediate and high-risk prostate cancer	October 4, 2016
	NCT02982941	I, Non-randomized	MacroGenics	25	Enoblituzumab	Enoblituzumab	Completed	Neuroblastoma, rhabdomyosarcoma, osteosarcoma, ewing sarcoma, wilms tumor, desmoplastic small round cell tumor	December 6, 2016
	NCT02381314	I, Non-randomized	MacroGenics	24	Enoblituzumab, ipilimumab	Enoblituzumab plus ipilimumab	Completed	Melanoma, non-small cell lung cancer	March 6, 2015
	NCT02475213	I, Non-randomized	MacroGenics	157	Enoblituzumab, pembrolizumab, MGA012 (anti-PD-1 monoclonal antibody)	Enoblituzumab plus pembrolizumab or enoblituzumab plus MGA012	Recruiting	Melanoma, head and neck cancer, non-small cell lung cancer, urothelial carcinoma	June 18, 2015
	NCT03406949	I, Non-randomized	MacroGenics	139	Orlotamab (MGD009, a humanized B7-H3 × CD3 DART® protein), MGA012 (anti-PD-1 monoclonal antibody)	Orlotamab plus MGA012	Recruiting	Advanced solid tumors	January 23, 2018

*In vivo* IPH2101 blockade of KIR resulted in better survival, showing preclinical evidence of efficacy in AML cells (acute myeloid leukemia) (93). Comparatively better clinical efficacy was evident in AML patients, with a median PFS of 7.7 months, RFS of 10.8 months, and OS of 12.7 months. These clinical outcomes were improved with increasing dose, but to a non-significant degree. Only OS showed significant increase with a dose of 1–3 mg/kg dose as compared to the previous dose of 0.3 mg/kg (27.9 vs. 11.8 months, *p* < *0.034*) (84). Safety and tolerability were acceptable, and AEs were mild and transient (83–85). An extension of this study revealed the role of lirilumab as maintenance treatment in elderly patients by prolonging survival and achieving satisfactory safety with repeated administrations (94). However, the results of an effiki trial revealed no significant difference among lirilumab regimens vs. placebo, casting doubts on lirilumab as a single agent in this setting (95). Lirilumab with full doses of azacytidine was well-tolerated in heavily pretreated/relapsed AML patients with high-risk features (96). The effectiveness and tolerability of lirilumab as monotherapy or combined with azacytidine in myelodysplastic syndrome (MDS) patients have been reported in a recent study (97). In a human model, a lirilumab and rituximab combination showed increased NK cytotoxicity in comparison to rituximab alone, in which autologous, EBV-transformed B cells were encountered by the individual's fresh primary NK cells, compared to a cell line model of anti-lymphoma therapy (98). In KIR transgenic and syngenic murine lymphoma models, this combination showed, *in vitro* and *in vivo*, an increased cytotoxicity mediated by NK cells and dependent on rituximab (99). These results provide and support a combined therapeutic approach of anti-KIR and anti-CD20 mAbs for clinical evaluation. More recently, a third member of this group of anti-KIR antibodies, IPH4102, a humanized anti-KIR3DL2 monoclonal antibody, has entered the clinical evaluation phase (Table 1). IPH4102, also termed Lacutamab, was declared safe in a phase I clinical evaluation of relapsed/refractory cutaneous T-cell lymphoma, with the most common adverse events including edema, fatigue, and lymphopenia. The clinical activity was encouraging as well, as 16 (36%) of the total of 44 patients achieved global overall response. Patients with relapsed/refractory cutaneous T-cell lymphoma with Sézary syndrome exhibited much better clinical response [15 (43%) of the 35 patients] (100). A phase II clinical trial (NCT03902184) is ongoing investigating IPH4102 as a single agent or in combination with chemotherapy in treating T-cell lymphomas.

## LIRs (ILTs)

Leukocyte immunoglobin-like receptor (LIR) or immunoglobin-like transcript (ILT), like KIR, belongs to the Ig superfamily and consists of activating as well as inhibitory receptors (53, 101, 102). Five inhibitory receptors (LIRB1-5) have been identified out of a total of 11 LIR members (103). Numerous immune cells (NK, T, B, and myeloid lineage cells that include macrophages and dendritic cells) variedly express these receptors (53, 101, 102). Among these, LIRB1 (ILT2) and LIRB2 (ILT4) recognize HAL-G as their main ligand, in addition to other ligands, leading to immunogenic tolerance (101, 104). LIRB1 or ILT2 is expressed on natural killer cells (36 ± 18% of normal NK cells), T cells, B cells, monocytes, subsets of DCs, and myeloid-derived suppressive cells (MDSCs), while ILT4 is expressed mainly on myeloid cells (21, 102). Therefore, ILT2 and HLA-G interaction could inhibit NK, T, and B cells' immune functions, thereby representing an immunotherapeutic target (105).

Various primary tumors and metastatic malignancies express HLA-G. It has also been regarded as an indicator of progressive disease and prognosis in various cancers (102, 106). Its expression has been associated with a decrease in NK functions in various cancers such as hepatocellular carcinoma (HCC), ovarian cancer, non-small cell lung carcinoma (NSCLC), glioma, and renal cell carcinoma (RCC) (107–111). Moreover, HLA-G surface expression or soluble HLA-G and/or its interaction with ILT2 has demonstrated inhibition of NK functions including cytotoxicity, cytokine production, and chemokine secretion (82, 102). Cytolysis by decidual NK cells was resisted by HLA-G-expressing target cells (112). HLA-G expression protected target cells against NK cell cytotoxicity in peripheral blood and NK cell lines (113, 114). ILT2 with HLA-G interaction has also shown inhibition of target cell-induced polarized IFN-γ production by NK cells (115). Chemotaxis and secretion of cytokines and chemokines by NK cells (CD56^bright^ and CD56^dim^) were differentially modulated by soluble HLA-G (sHLA-G). Secretion of CCL2 by both CD56^bright^ and CD56^dim^ NK cells and CCL2, CCL8, and CXCL2-CXCL3 by CD56^dim^ NK cells from peripheral blood were upregulated by sHLA-G (116). HLA-G1/ILT2 interaction was also shown to mitigate MICA/NKG2D activation by inhibiting NK cytotoxicity (117). In gastric cancers, HLA-G/ILT2 interaction led to inhibition of infiltrating NK cell proliferation and cytotoxicity (118). HLA-G plasma expression in B-cell malignancies, however, has been shown to inhibit malignant B cell proliferation due to their expression of ILT2 surface receptors, which is lacking in solid tumor cells (102, 119). This may have been the reason for the failure of LIR-1 blockade in enhancing the cytotoxicity of the NK cells against MM cells (120). However, a dual blockade of LIR-1 and NKG2A was able to increase the cytotoxicity of KIR-negative NK cells (21). These findings suggest that a dual role is played by HLA-G-ILT2 interaction: the role of the classical inhibitory checkpoint in solid malignancies and the role of disease progression in hematological malignancies due to ILT2 expression on the hematological malignant cells. Hence, blocking this checkpoint with antibodies can be considered a possible potential target in solid cancers. Further exploration is needed to confirm its role in hematological cancers. Lenalidomide has also shown immunomodulation of NK cells and leukemic cells in CLL (Chronic Lymphocytic Leukemia). ILT2 expression is increased in CLL on NK cells and decreased on leukemic cells. The immunomodulatory effects of lenalidomide increase ILT2 expression on leukemic cells and partially recover its ligand (HLA-E) expression as well. Moreover, NK cell activation and proliferation were also increased. ILT2 blockade further potentiated NK cell activation and proliferation in this setting (121) (Figure 3).

## NKG2A and CD94

NKG2A (also known as CD159) & CD94, a heterodimer inhibitory receptor of the C-type lectin family, recognizes a non-classical MHC-I molecule, HLA-E, as ligand (22). CD94-NKG2A and its HLA-E ligand are non-polymorphic. HLA-E^*^0101 and HLA-E^*^0103 represent the only two alleles exhibited by HLA-E in worldwide populations (122, 123). Almost 50% of the NK cells in the peripheral blood express CD94/NKG2A, primarily those that do not express inhibitory KIR. The co-expression of CD94/NKG2A with other inhibitory receptors of different specificity also exists. In addition, γδ and CD 8^+^ T cells also express CD94/NKG2A (21, 124). Ligation of NKG2A & CD94 to HLA-E expressed on normal cells suppresses signaling activation, thereby avoiding the destruction of normal bystander cells (125).

Tumor cells (hematological as well as solid tumors), in order to avoid killing by NK cells, have shown upregulation of HLA-E expression. In various cancers, poor prognosis has been associated with HLA-E upregulation, including colorectal (126, 127), ovarian (122), gynecologic cancers (123, 128), liver (129), glioblastoma (130, 131), Hodgkin lymphoma (132), chronic lymphocytic leukemia, esophagus, gastric, pancreas, colon, kidney, head and neck, lung and melanoma (124). Blocking of the CD94/NKG2A receptor with an antibody could be used as a therapeutic strategy. Hence, an antibody against CD94/NKG2A (IPH2201-Monalizumab), developed by Innate Pharma, has been employed in various trials (17) (Table 1). *In vitro* and *in vivo* findings have suggested the application of humanized anti-NKG2A antibody against hematologic malignancies to be safe and effective (133). Improvement of NK-cell dysfunction by monalizumab in chronic lymphocytic leukemia has been shown *in vitro* (134). Monalizumab was well-tolerated (IV or SC dosing up to 10 mg/kg) as monotherapy in gynecologic malignancies with no reported DTLs or SAEs. This ongoing trial of heavily pretreated cohorts revealed a stabilized disease in 41% of evaluable patients (128).

A transition from monotherapy to a combined therapeutic approach is on the rise in the field of immune checkpoint inhibitors, mainly because some of these receptors are heavily expressed on several innate and adaptive immune cells simultaneously, as well as due to intercellular interaction and interdependence. Monalizumab is being evaluated in combination with durvalumab, cetuximab, and ibrutinib. Various solid cancers that express HLA-E have infiltrating CD8^+^ T, NK, and NKG2A^+^ immune cells (124). These infiltrating NKG2A^+^ NK cells and CD8^+^ T cells have demonstrated enhanced NK- and T-cell responses upon receptor blocking (135). It has been reported that PD-1 is coexpressed along with NKG2A in tumor-infiltrating NK cells and CD8^+^ T cells. *In vitro* and *in vivo* blocking of both NKG2A/HLA-E and PD-1/PD-L1 pathways with antibodies have shown complete response rate (124, 135, 136). A combination of monalizumab and durvalumab has shown clinical efficacy and a manageable toxicity profile, with no DTLs, as suggested by preliminary data in patients with heavily pretreated metastatic microsatellite colorectal cancer (137).

*In vitro* findings have revealed the additive efficacy of anti-NKG2A antibody in combination with other immune-oncology treatments such as anti-EGFR (cetuximab) in an SCCHN cell line and anti-CD20 (obinutuzumab) in cocultures with B cell lines expressing MHC class I (135). The induction of ADCC by cetuximab and the possible inhibition of cetuximab-mediated cytotoxicity by CRC (colorectal cancer)-expressed HLA-E provided the basis for a combined therapeutic approach (135, 138, 139). Preliminary assessment of the safety and efficacy of a monalizumab and cetuximab combination in head and neck squamous cell carcinoma (SCC) that was previously treated, recurrent, and/or metastatic revealed a 27.5% ORR (objective response rate), a 5-month median PFS (progression-free survival), and a 10-month median overall survival (OS). This is an encouraging outcome if compared to historical records of the efficacy of cetuximab alone from previous studies (ORR 12.6%, PFS 2.3 m, OS 5.6 m). The adverse events profile of the combined approach was similar to that of cetuximab alone (140). Recent *in vivo* analysis has demonstrated that vaccine therapy efficacy is hampered by the induction of NKG2A on CD8^+^ T cells and that blocking of the NKG2A receptor leads to improved efficacy of vaccine therapy (141). Overall, blocking of NKG2A represents an exciting therapeutic approach, and in particular, its combination with other immune-oncology therapeutic agents is the way forward and permits further exploration.

## Tigit and CD96

TIGIT (T cell immunoreceptor with immunoglobulin and ITIM domains) is an immune inhibitory receptor expressed on NK and T cells such as activated NK, T, mT (memory T cells), fTh (follicular T helper cells), and regulatory T cells (Tregs) (56, 142, 143). CD96, a member of the same immunoglobulin superfamily, has a similar inhibitory role but with lower binding affinity for the ligand CD155 as compared to TIGIT. CD226 is an activating receptor that competes in binding to CD155 with TIGIT and CD96 (144–146). CD155 (mainly) and CD112 serve as ligands for TIGIT & CD96 to bind in order to inhibit T cell- and NK cell-mediated immunity (55, 57, 143, 147–151). It certainly is an important receptor mediating innate as well as adaptive immune responses. CD155 is a transmembrane glycoprotein, also known as poliovirus receptor (PVR) as it was first identified as a poliovirus entry receptor. PVR (CD155) is a member of the immunoglobulin superfamily as well as being the fifth member of the nectin-like molecule family and is therefore also known as necl-5 (152). It is barely expressed in normal human tissues, but many tumor cell lines and primary malignancies highly express PVR (54, 153). Of the functions performed by PVR, immunoregulation through its interaction with inhibitory receptors TIGIT and CD96 and activating receptor CD226 is of particular interest. Various cancers have shown upregulation of CD155 with corresponding upregulated NK and T cell expression of TIGIT and CD96 in order to evade anti-tumor immunity by eliciting T cell or NK cell inhibition. Preclinical evidence supports the idea of blockading this checkpoint for the activation of NK cell-mediated antitumor immunity (56, 145, 151, 154). Clinical translation is in its preliminary stages (148).

NK, T (effector and memory), and regulatory T cells express TIGIT. So far, TIGIT blockade has been evaluated, primarily in hematologic tumors, in reference to its expression on T cells, including CD8^+^, CD3^+^, and regulatory T cells (154, 155). TIGIT blockade in MM is mostly explored in relation to CD8^+^ T cells. Multiple myeloma tumor cells show upregulation of CD155 ligand. Immunosurveillance and therapy of multiple myeloma were demonstrated to be dependent on CD226 experessed by NK and T cells (156). TIGIT blockade prevented the T-cell exhaustion mechanism responsible for myeloma escape after stem cell transplantation (157). CD8^+^ T cells expressing high levels of TIGIT on their surfaces were associated with multiple myeloma progression. Immunity was enhanced against multiple myeloma by its blockade in mice as well as in humans (158). Anti-TIGIT therapy could be tested for its clinical efficacy as monotherapy or combined with other therapeutic agents for the treatment of multiple myeloma patients after the failure of a single checkpoint agent targeting PD-1 receptors (159).

Poor clinical outcomes for patients with acute myeloid leukemia (AML) and CD8^+^ T cell exhaustion were associated with TIGIT (160). AML cells lysis was significantly augmented by T cells through *in vitro* blockade of TIGIT and PVR or PVRL2 interaction alone or in combination with the BiTE® antibody construct AMG 330 (161). Patients with higher TIGIT expression in the bone marrow (BM) after alloSCT had a significantly lower incidence of grade II–IV acute graft vs. host disease (aGVHD) (*p* = 0.048), shorter PFS (*p* = 0.024), and shorter OS (*p* = 0.046). Higher TIGIT expression also had lower NK cell counts in the BM after alloSCT, suggesting that TIGIT might play a crucial role in the GVL effect and GVHD to control NK cell activity and proliferation after alloSCT. Based on these observations, it was suggested that TIGIT could be a prognostic predictor after alloSCT and that its blocking could be a potent immunotherapeutic strategy to intensify the graft-vs.-leukemia effect after alloSCT in AML patients (162). Hodgkin and Reed–Sternberg (HRS) cells or Tregs are involved in the inhibition of Th1, CD8^+^ T cells, and NK cell activity through PD-1 (163). Variable expression of PD-1 and TIGIT was observed on CD3^+^ T cells in patients with Hodgkin lymphoma, suggesting that TIGIT blockade alone or in combination with other drugs might be used as a potential therapeutic target. However, further evaluation is required (164). As PD-1 or TIM-3 are co-expressed by TIGIT-positive T cells, targeting TIGIT could be an additional mechanism to avert exhaustion of T cells in B-cell Non-Hodgkin Lymphoma (B-NHL) (155). Though HL and NHL express TIGIT on T cells, its expression on NK cells and relevant therapeutic application in this group of patients have not been evaluated so far.

Several tumor mice models have shown TIGIT upregulation on CTL and NK cells during tumor progression. *In vivo* tumor growth of inoculated B16/F10 was delayed by TIGIT deficiency, while exhaustion of effector cells (CTL and NK cells) targeting tumor was reversed by *in vivo* TIGIT blocking in combination with *in vivo* Flt3L overexpression by gene delivery and improved overall survival by significantly suppressing pre-established B16/F10 tumor growth and metastasis (151). CD155 expression was associated with prognosis in human pancreatic cancer. This expression was also inversely correlated with tumor-infiltrating lymphocytes and positively correlated with VEGF expression and angiogenesis (165). However, TIGIT expression was found to be similar between pancreatic cancer patients and healthy controls, while CD226 and CD96 were downregulated (166). There seems to be a dysregulation of the TIGIT/CD96/CD226/CD155 pathway involving NK cells in pancreatic cancer patients, warranting further *in vitro* and *in vivo* evaluation.

NK and CD8^+^ T-cell functions were suppressed by TIGIT-intrinsic expression, thereby helping tumor (colorectal) growth *in vivo* (167). TIGIT was associated with exhaustion of NK cells in tumor-bearing mice and colonic cancer patients, while this exhaustion was reverted by its blockade, thereby eliciting potent antitumor immunity. The presence of NK cells was important for the therapeutic efficacy of TIGIT and/or PD-L1 blockade or dual blockade of both the checkpoints, as NK cell absence was associated with a lower frequency of IFNy- or TNF-secreting TILs (CD8^+^) and a higher frequency of PD-1-expressing TILs (CD8^+^) (167). NK cells constitute 25–50% of liver lymphocytes, which shows their importance for liver immunity. Moreover, the survival and prognosis of HCC patients were positively correlated with NK cell numbers in blood and tumor tissue (168, 169). Tumor progression of the HCC patients was associated with dysfunction of the tumor-infiltrating NK cells, primarily the CD11b-CD27-NK subsets (170). Sun et al. identified exhausted tumor-infiltrating CD96^+^ NK cells and found their expression to be correlated with poor clinical outcome for HCC patients. NK cell exhaustion was reversed when CD96-CD155 interaction or TGF-β1 was blocked (171).

There are conflicting reports about the effectiveness of TIGIT blockade against metastatic disease. TIGIT is considered not to have any effectiveness against metastasis formation, as the number of lung nodules found was comparable in *TIGIT*^−/−^ mice and wild-type mice after intravenous injection with B16 melanoma cells (146). *In vivo* TIGIT signaling blockade improved overall survival by significantly suppressing pre-established B16/F10 tumor growth and metastasis (151). However, this effect was achieved in combination with Flt3L overexpression *in vivo* by gene delivery. On the other hand, several studies have reported CD96 efficacy against metastatic disease. *CD96*^−/−^ mice had fewer lung metastases as compared to wild-type mice after intravenous injection with B16 melanoma cells (146). Experimental metastases were inhibited in three different tumor models with monoclonal antibody blockade of CD96. This suppression by CD96 mAb was NK cell-, CD226-, and IFN-γ-dependent but independent of activating Fc receptors. Furthermore, CD96 blockade enhanced metastatic control in the absence of TIGIT (172).

Recently, increased emphasis has been placed on the combination of checkpoint inhibitors in order to achieve a synergistic effect. Improved survival was reported in tumor-bearing mice with dual targeting of PD-1 and TIGIT by enhancing CD8^+^ T-cell activation (173). Dixon et al. also reported that dual blocking of TIGIT and PD-1 in an MC38 colon carcinoma model leads to a synergistic anti-tumor effect, resulting in complete tumor regression (174). Dual blockade of TIGIT and PD-1 in melanoma patients synergistically increased the proliferation, degranulation, and cytokine secretion of tumor-infiltrating and tumor antigen-specific CD8^+^ T cells, demonstrating a potential for dual blockage (175). Hong et al. suggested that both PD-1 and TIGIT may serve as potential targets for the treatment of RCC as well (176). In GBM patients, such dual blockade has also shown to enhance anti-cancer immunity as well as survival (177). Though these studies have reflected the efficacy of dual checkpoint blockade in various cancers by exploring the role of T cells, there are some studies that have shown efficacy of dual checkpoint to be dependent on NK cells as well. Anti-TIGIT plus anti-PD-L1 blockade prevented the exhaustion of NK cells in tumor-bearing mice and colon cancer patients (167). On the other hand, a combination of Anti-CD96 with doxorubicin chemotherapy, anti-CTLA-4, or anti-PD-1 has shown more efficacy in inhibiting experimental metastases in three different tumor models (172). Exhausted NK cells from patients with bladder cancer (BC) showed upregulation of TIM-3 and TIGIT in both the periphery and tumor (178). In fact, the role of TIGIT and CD96 in NK cell exhaustion in various cancers is under investigation, and further revelations are needed to establish their potential for targeting either as monotherapy or in combination with other checkpoints.

## Siglec-7/9

Sialic acid-binding Immunoglobulin-like lectins (Siglecs) are immunomodulatory sialic acid-binding receptors belonging to the I-type lectin family (179). Siglecs are expressed on various immune cells that include immune cells of both lymphoid and myeloid origin, namely neutrophils, eosinophils, monocytes, macrophages, NK cells, DCs, mast cells, and B and T cells (58, 180–183). Siglecs shows variety in two properties: their expression and their specificity for sialic acid-containing ligands (184). Most of these Siglecs are inhibitory receptors, such as Siglec-2, Siglec-3, Siglec-5, Siglec-6, SIglec-7, Siglec-8, Siglec-9, Siglec-10, and Siglec-11 (58). Of the inhibitory Siglecs, Siglec-7 and Siglec-9 are reported to be expressed on human NK cells (185–187). Similar to classical NK cell inhibitory receptors NKG2A/CD94 and KIRs, inhibitory Siglecs also contain one or more ITIMs and ITIM-like motifs in their cytoplasmic tail. Upon ligation, ITIMs are phosphorylated by Src family kinases, recruiting and activating SRC homology 2 (SH2)-domain-containing proteins, mainly the tyrosine phosphatases SHP1 and SHP2 or the suppressor of cytokine signaling 3 protein (SOCS3) (58).

Changes in sialic acid, a nine-carbon sugar, have been related to cancer (188, 189). Apart from aberrant tumor cell surface expression, sialic acid alterations and the amount or density of sialic acid have been associated with tumorigenesis and cancer progression. These include hypersialylation, xenosialylation (uptake of Neu5Gc), and sialic acid alterations, including C5-hydroxyl modification of sialic acid [which generates 2-keto-3-deoxy-D-glycerol-D-galac- to-nononic acid (KDN)] and O-acetylation of sialic acid (particularly 9-O-acetylation) (189). Hypersilaylation has been associated with several cancers such as oral cancer, RCC (renal cell carcinoma), HNSCC (head and neck squamous cell carcinoma), breast cancer, prostate cancer, and colon cancer (184). KDN changes have been associated with ovarian cancer (190) and carcinoma of the head and neck (191), while O-acetylation of sialic acid has been reported in colorectal cancer (192, 193).

Siglec-sialic acid interactions are involved in the modulation of immune tolerance and can be targeted for eliciting anti-tumor immunity (184). Targeting some of these inhibitory checkpoints (anti-Siglec-2; Inotuzumab ozogamicin and anti-Siglec-3; Gemtuzumab ozogamicin) with antibodies conjugated to a cytotoxic agent have already been tested for their clinical efficacy (182, 183, 194). Human NK cells mostly upregulate Siglec-7 and Siglec-9. Furthermore, in cancer, peripheral NK cells also upregulate Siglec-9, primarily on CD56^dim^ CD16^+^ NK cells (58, 187). Blocking of Siglec-7 and Siglec-9 with Fab fragments increased the *in vitro* cytotoxicity of NK cells against tumor cells (K562). In an *in vivo* model of immunodeficient mice with transferred human NK cells and human tumor cells, killing of tumor cells was mediated by the sialoglycan-dependent NK cell inhibition (187). High and sustainable cytotoxicity against leukemia cells was shown by a Siglec-7(negative) phenotype of developed NK-92MI cell line (195). Recruitment of Siglec-7 was essential for inhibition of human NK cell activation by artificially increased sialylated glycans on cancer cells. The susceptibility to NK cell killing of various remodeled tumor lines of breast, brain, colon, liver, or lymphoid tissue (Siglec-7-abundant tumor cell lines) was increased after sialidase treatment. Siglec-7 engagement provided protection against NK cell killing by inhibiting both antibody-dependent and antibody-independent cytotoxicity. Cancer cells expressing ligands for Siglec-7 can protect themselves from the innate immune response as well as therapeutically relevant ADCC (196). *In vitro* fusion of sialidase to antibodies targeting HER2 enhanced the HER2^+^ tumor cell killing by NK cells. This enhanced NK cell-mediated killing was possible through cutting off the sialic acid ligands by sialidase, specifically the ones bound by Siglec-7 and Siglec-9. This demonstrates that glycocalyx-selective desialylation of tumors could make them more susceptible to ADCC with such antibody-sialidase conjugates (197). Siaglec-7 show high preference for engagement with cis sialic acid residues on the surface of human NK cells results in masking of these siaglec-7 (198). Siglec-9 has also shown such self-sialic acid interactions (188). Engineered overexpression of α2,8-linked disialic acids on tumor cells showed a slight reduction in NK cell cytotoxicity, which was intensified after treatment of NK cells with sialidase, which cleaves the *cis* ligands from the NK cell surface. α2,8-linked disialic acids are overexpressed by the GD3 ganglioside produced at high levels in cancers like melanoma, making them susceptible to such sialidase-treatment-driven NK cell cytotoxicity (199).

*In vitro* NK cell cytotoxicity was potently enhanced by high-affinity anti-Siglec-9 antibodies through blocking sialic acid expressed on tumor target cells. These antibodies targeting Siglec-9 also had improved anti-tumor response, induced by the NKG2A blockade (188). Siglec-9 is also upregulated on tumor-infiltrated CD8^+^ T cells in NSCLC, ovarian, and colorectal cancers (181, 188). A subset of intratumoral effector memory CD8^+^ T cells in melanoma have also shown Siglec-9 upregulation and suppression by its engagement through phosphorylating SHP1 (200). *In vitro* and *in vivo* targeting of the sialoglycan-SAMP/Siglec pathway resulted in increased anticancer immunity. Other inhibitory receptors such as PD-1 were also co-expressed by T cells expressing Siglec-9, hinting at a potential for co-inhibition (181). The multi-mode action of Siaglec-9 is apparent from the fact it is expressed on various types of immune cells (188). These data lend support to the idea that anti-Siglec-7 and anti-Siglec-9 blocking antibodies should be developed for cancer immunotherapy, potentially in combination with other immune checkpoint inhibitors.

## LAG-3

LAG-3 (Lymphocyte Activation Gene-3) is also a member of the immunoglobulin superfamily receptors with inhibitory properties. LAG-3 was discovered as an upregulated molecule on the surface of activated CD4^+^ T cells, CD8^+^ T cells, and NK cells (201). In addition to these cells, Lag-3 is expressed on several other immune cells including TILs, regulatory T cells, iNKT cells, B cells, and DCs (68, 202–207). It recognizes MHC class II molecules as ligands and shares structural similarity with CD4 molecules but binds to MHC-II molecules with greater affinity than CD4 (201, 208, 209). LSECtin, expressed in liver and several other tumors and a member of the DC-SIGN family, has also been described as a potential ligand for LAG-3-expressing immune cells (210). Engagement of LAG-3 inhibits T cell effector function and is involved in T-cell exhaustion (211–213). It also promotes the suppressive activity of regulatory T cells (203, 214). Blocking of LAG-3 has been shown to induce improvement in T-cell functions (212). Relatlimab is an anti-LAG-3 monoclonal antibody being investigated in several ongoing clinical trials, either alone or combined with PD-1 blockade, in various cancers (215). LAG-3 and PD-1 have shown synergism in T-cell functional regulation to promote tumor immune escape (216).

Though LAG-3 is expressed on NK cells, its role in the regulation of NK cells has not been well-established. Knockout of the LAG-3 gene in a mouse model resulted in the inability of NK cells to kill certain tumor targets. However, this deletion had no effect on MHC class I-mismatched cytolytic activity (217). Human NK cells, on the other hand, showed an opposite result. Antibodies blocking the LAG-3 pathway were unable to induce human NK cell cytotoxicity. Soluble Lag-3, capable of binding to MHC-II molecules, also had no effect on human NK cell killing capability. However, in this research, cytokine production was not investigated (218). In patients with HIV, viral control was associated with low LAG-3 expression on NK cells along with other inhibitory molecules (219). Wiskott-Aldrich Syndrome protein (WASp) deficiency is associated with a high susceptibility to cancer, most probably due to impairment of the anti-cancer capacity of NK cells and DCs (220, 221). WASp knockout NK cells showed an association of cellular exhaustion and NK cell memory with enhanced LAG-3 expression (222). There seems to be an evident association; however, the direct impact of LAG-3 on NK cell functions and underlying mechanisms needs further investigation. In contrast to NK cells, its regulation of NKT (Natural Killer T cell) functions has been well-reported. In patients with chronic HIV, exhaustion of iNKT cells and reduction in IFN-γ production were associated with elevated expression of LAG-3 (204). The LAG-3 signaling pathway, through arresting the cell cycle in the S phase, also down-modulated the proliferation of activated CD1d-restricted NKT cells (223).

A soluble recombinant LAG-3-Ig fusion protein, Eftilagimod alpha (IMP321), has been used as an immunological adjuvant for vaccination against various infections and cancer. It has also been applied as monotherapy or combined with chemotherapy in cancer (224). IMP321 was able to induce NK cells to produce cytokines (IFN-γ and/or TNF-α) in healthy individuals (52 of 60 donors) and, to a lower extent, in 21 untreated metastatic cancer patients in an *ex vivo* short-term experiment (225). In metastatic renal cancer patients, IMP321, in a dose-escalation study (P003), induced NK cell activation as monotherapy (226). IMP321 with standard chemotherapy was associated with enhanced activation of NK cells for several months in breast cancer patients (227). Hence, LAG-3 has the potential to activate T cells as well as NK cells. Hence, it can be further explored as a potential target for checkpoint inhibition. Furthermore, CD56^bright^, CD16^−^, and CD62L^+^ NK cells were identified as the dominant subset of cytokine-induced memory-like (CIML) NK cells, with sustained expression of NKG2A involved in inhibition of killing of the HLA-E-positive target cells in a recent study on CIML NK cells. A minor CIML NK cell subset, KIR^+^ and NKG2C^+^, was shown to express LAG-3, suggesting CIML NK cells as a potential target for dual checkpoint inhibition (228).

## TIM-3

A co-inhibitory receptor, TIM-3 (T-cell immunoglobulin and mucin domain 3), recognizes galectin-9 as ligand, which is upregulated in various cancers and chronic infections (68, 229–234). In addition, the TIM-3 variable IgV domain has also been reported to bind to other ligands, such as HMGB1 (high mobility group protein B1 proteins), Ceacam-1 (carcinoembryonic antigen cell adhesion molecule 1), and PtdSer (phosphatidylserine) (229, 230). TIM-3 expression is diverse, encompassing several types of immune cells, including CD4^+^ T cells, CD8^+^ T cells, regulatory T cells, B cells, NK cells, NKT cells, and myeloid cells (230, 235). TIM-3 engagement with its ligands induces immune tolerance by exhausting T cells as well as NK cells (232, 236–238). This pathway upregulation is associated with exhaustion of T and NK cells in various chronic infections as well as cancers, making TIM-3 a negative regulator of T and NK cell immunity (232, 234). Correspondingly, its blockade has reversed T or NK cell dysfunction (238–242). Co-expression of TIM-3 and PD-1 was involved in mediating the exhaustion of CD8^+^ T cells in various cancers and chronic viral infections (243–248). Studies have revealed a reversal of T-cell exhaustion and reduction in tumor growth with TIM-3 and/or PD-1 blockade together (245, 247, 249, 250). Antibodies for TIM-3, such as Sym023, Cobolimab, LY3321367, BGB-A425, and MBG453, in combination with several anti-PD-1/PD-L1 antibodies, are under clinical investigation for their efficacy against various cancers (Table 1).

TIM-3 expression on NK cells has several aspects to it. It has been regarded as a maturation, activation, and prognostic marker. TIM-3 is highly expressed in the resting CD56^+^/CD3^+^ NK-cell population as compared to the CD56^+^/CD3^+^ NKT and CD56^−^/CD3^+^ T-cell populations (251). A fraction of the mature CD56^dim^CD16^+^ NK cell subset in blood from healthy adults displayed TIM-3 expression, while its expression was heterogeneous in the immature CD56^bright^CD16^−^ NK-cell subsets. Moreover, several cytokines (IL-12, IL-15, and IL-18) strongly induce TIM-3 expression, primarily in the immature CD56^bright^ NK cells (251, 252). IL-12 and IL-18-induced activation and IL-15-induced maturation of NK cells are the main cause of TIM-3 expression in these cells, identifying TIM-3 expression in NK cells as a marker for the activation and differentiation or both (241, 251). Upregulation of TIM-3 in the peripheral NK cells is observed in several cancers, namely gastric cancer (253), lung adenocarcinoma (238), advanced melanoma (242), and bladder cancer (178) leading to NK cell exhaustion. Increased levels of TIM-3 in NK cells with tumor growth shows TIM-3 expression to be a prognostic biomarker (178, 238, 241, 242, 253). Tumor-infiltrating NK cells in patients with GIST (gastrointestinal stromal tumors) and bladder cancer were also revealed to express TIM-3 (178, 254). Similar to TIM-3 and PD-1 co-expression in T cells, exhausted tumor-infiltrated NK cells have also shown detectable co-expression in MHC-I-deficient tumors (255). However, patients with GIST lacked PD-1 co-expression in TIM-3^+^ tumor-infiltrating NK cells (254).

TIM-3 has been described as an activation marker and activation limiter of NK cells, since its engagement to its ligand (Gal-9) and blockade have shown contrasting results (256). TIM-3 engagement with Gal-9 was able to induce IFN-y production by NK cells with no effect on cytotoxic ability (251), and anti-Gal-9 antibody blockade of TIM-3 reduced the production of IFN-γ by NK cells from healthy donors in response to primary AML blasts (231). TIM-3 blockade on IL-15-stimulated NK cells also resulted in cytotoxicity reduction for two PCC lines (human pancreatic cancer cell), Mia-Paca-2 and Capan-2, though a PSC line panel (human pancreatic stellate cell) showed no significant difference in cytotoxicity (257). *In vitro*, Gal-9 downregulated TIM-3 on NK cells and triggered NK cell activation in HIV-1 infection; however, a subset of these NK cells (immature CD56^bright^ NK cells) were not affected by this decrease in TIM-3 expression; rather, an increase in the surface expression of TIM-3 was witnessed with a concomitant increase in Gal-9 expression during late primary HIV-1 infection (258).

TIM-3 upregulation on NK cells and its association with NK cell exhaustion and dysfunction has been reported in a number of studies, defining TIM-3 as a negative regulator. TIM-3 engagement with agonist antibody or Gal-9-expressing target cells revealed significant suppression of NK cell-mediated cytotoxicity in NKL, a human NK-cell line, or primary human peripheral blood NK cells from PBMCs (252). TIM-3 expression on NK cells was also increased in chronic HIV infection associated with a dysfunctional phenotype of NK cells (237). During early pregnancy, an upregulation of TIM-3 in NK cells was observed, leading to inhibition of NK cell cytotoxicity toward trophoblast in a Gal-9 dependent pathway (259). NK cell expression of TIM-3 receptors was also upregulated by chronic hepatitis B infection, causing subsequent suppression of NK cell function, and was rescued by TIM-3 blocking (260). TIM-3 blockade also had increased NK cell cytotoxicity against K562 target cells (261). Blockade of the TIM-3 pathway in patients with several cancers, such as advanced melanoma and lung adenocarcinoma, restored NK cell cytotoxicity. TIM-3 blockade enhanced effector function in peripheral NK cells from bladder cancer patients (178).

It has been suggested that, when the expression of TIM-3 is upregulated, it initially enhances NK cell cytotoxicity, and that chronic activation leads to overexpressed or dysregulated TIM-3 expression, resulting in a subset of dysfunctional or exhausted NK cells (256, 258). TIM-3 unselective binding to its ligands, Gal-9 inhibition of NK cells in a TIM-3-independent manner (236), and Gal-9 downmodulation of TIM-3 in chronic HIV patients (258) may account for some of these shortcomings upon further investigation. The ineffectiveness of TIM-3 blockade in intra-tumor NK cells also reveals the role of tumor-specific factors, and its potentiation after activation with IL-15 suggests that inflammatory cues also have a role to play (178).

## CD200R

CD200R is another inhibitory receptor expressed on T, B, NK, and myeloid cells (262–264). It recognizes CD200 as its ligand, which is expressed on various normal tissues such as the central nervous system, retina, hair follicular cells, vascular endothelial cells, and thymocytes, as well as activated T, B, and DCs, in addition to its expression on various tumors (265). CD200 is considered as a marker of tumor progression since it is overexpressed on various cancers of both hematopoietic and non-hematopoietic origin, such as acute myeloid leukemia, multiple myeloma, hairy cell leukemias, B cell chronic leukemias, melanoma, and ovarian, rectal cancer, and bladder cancer, and its expression is associated with the worst prognosis (266–274). Moreover, CD200 expression can also be induced on cancer cells (275, 276). In fact, cancer cell expression of CD200 had no effect on suppression of anti-cancer responses by CD200–CD200R signaling (277). Therefore, CD200 blockade represents a potential therapeutic option not restricted to the treatment of CD200-expressing tumors (265). The inhibitory CD200–CD200R pathway appears to inhibit the effector functions of T cell in an indirect manner via the regulation of macrophages and DCs. Hence, tumor growth could be inhibited by blockade of CD200–CD200R interaction, lending support to the idea that antagonistic CD200 or CD200R antibodies are an option in cancer treatment (278). Samalizumab (a humanized Anti-CD200 antibody) was well-tolerated and demonstrated changes in CD4-positive T cells and CD200-positive B-CLL in a dose-dependent manner as well as inducing a dose-dependent linear increase in serum AUC and modest Th1 cytokine responses (279).

In the case of NK cells, there is evidence of direct involvement of the CD200–CD200R inhibitory pathway resulting in the suppression of NK cells. In AML patients, NK cell anti-tumor responses were suppressed by the overexpression of CD200, thereby increasing risk for relapse in these patients (280). NK cell subpopulations in AML patients expressed CD200R, suggesting NK cell suppression from CD200–CD200R interaction. Moreover, in patients with CD200hi, antibody blocking CD200 restored NK cell activity. These data suggest that CD200–CD200R interaction is directly responsible for NK cell suppression in AML patients (264). These are the only studies directly indicating that target cells expressing CD200 could inhibit the cytotoxic as well as the IFNγ-producing activities of NK cell. Liu et al., while investigating the CD200 signaling part in CD200^+^ melanoma growth and metastasis restriction, revealed a significantly reduced number of NK cells in livers undergoing metastatic tumor growth that were deficient in CD200. However, how deficiency in CD200 could affect local NK responses in liver remains to be explained (272). It suggests that the CD200–CD200R checkpoint is an appreciable target for checkpoint blockade in hematologic and solid tumors. Since NK cells have been targeted in relation to other checkpoint receptors in AML and multiple myeloma, such as KIR and NKG2A, a combination of checkpoint targets could be validated in this group of patients.

## CD47

CD47, also referred to as integrin-associated protein (IAP), is a glycoprotein of the Ig superfamily that is expressed ubiquitously (281). It was first discovered on leukocytes as a membrane protein taking part in signaling mediated by β3 integrin. It is a transmembrane protein that also interacts with thrombospondin-1 (TSP-1) and signal regulatory protein-alpha (SIRPα), in addition to integrins (282–285). Of the functions performed by CD47, its engagement with SIRPα and thrombospondin-1 has established its role as an inhibitory receptor involved in immune evasion by cancers through inhibition of phagocytosis, antigen presentation, and T/NK cell inhibition (286–289). Consequently, targeting these signaling pathways with antibodies has revealed the therapeutic candidacy of this checkpoint (283, 289–292).

CD-47 has been disclosed to play an inhibitory role in NK cell-mediated anti-viral or anti-tumor cytotoxicity. Deficiency in CD47 was shown to impair the immune responses of NK cells to LCMV infection (293). NK cell cytotoxicity was associated with CD47 expression on HNSCC cell lines. High CD47-expressing HNSCC cell lines exhibited NK cytotoxicity at lower levels compared to those with low expression of CD47. Pretreating cells with neutralizing MHC-1 or anti-CD47 antibodies led to an increase in NK cell cytotoxicity against HNSCC cell lines (289). This study did not reveal an associated CD47-receptor ligating partner. Both ligands SIRPα and TSP-1 have been associated with NK cell-mediated cytotoxicity. In immunocompetent syngeneic mice, RCC or melanoma cell tumor formation was markedly suppressed by anti-SIRPα antibody through blockade of CD47 interaction. Selective depletion of NK cells greatly attenuated the antitumor effect of anti-SIRPα Ab, in addition to macrophages and CD8^+^ T cells (294). However, *in vitro* killing of the tumor cells by NK cells was not inhibited by the same antibody, suggesting CD47 function in NK cells to be independent of SIRPα. In a similar fashion, thrombospondin-1 was indicated in inhibiting early NK cell proliferation and enhancing late expansion, but the role of CD47 was not identified (295). Therefore, there is still much to be unraveled in this regard. Nonetheless, attacking CD-47 with antibodies is worth exploring, not only in the context of macrophages, dendritic cells, and T cells but also NK cells. Dual blockade of CD47 in combination with PD-L1 has also been explored and has shown to enhance immunotherapy against circulating tumor cells (296). Magrolimab (Hu5F9-G4), an anti-CD47 antibody, is under investigation in several phase I and II clinical trials in various combinations with other agents such as rituximab, cetuximab, azacitidine, acalabrutinib, and atezolizumab (Table 1).

## B7-CD28 Family Receptors

The B7-CD28 family of ligands and receptors play important roles in T-cell co-stimulation and co-inhibition. Recognition of their role in relation to NK cells in on the rise, with accumulating evidence. Primarily, CTlA-4 and PD-1 checkpoints, just like T cells, have been implicated in NK cell dysfunction in various cancers. Newer checkpoints from this family, including B7-H3, VISTA (PD-1H), and B7-H7, are being discovered to show inhibition of T and NK cell-mediated cytotoxicity (297).

## CTLA-4

CTLA-4 (Cytotoxic T lymphocyte-associated antigen 4), a co-inhibitory receptor, is expressed on several immune cells, such as activated T lymphocytes (CD4^+^ T cells & CD8^+^ T cells), regulatory T cells, tumor-infiltrating NK cells, and splenic Kit^+^ CD11b^−^ NK cells in tumor-bearing mice, and is induced on mouse NK cells upon IL-2 stimulation (298–300). CTLA-4 competes with CD28, a costimulatory receptor, for ligands B7-1 (CD80) and B7-2 (CD86) on cancer cells or antigen-presenting cells. CTLA-4 is well-established as a negative regulator of T-cell activation and controller of peripheral T-cell tolerance and autoreactivity (298, 301–308). Blockade of CTLA-4 with antibody (ipilimumab) has improved T-cell function in various cancers (309).

Previous studies have indicated CTLA-4/CD28/CD80/CD86 pathway involvement in NK cell-mediated cytotoxicity. *In vivo*, CD28 triggering the proliferation of NK cells, their cytotoxicity, and secretion of cytokines has been described in a number of studies (310, 311). Ligands B7-1 and B7-2 on cancer cells both also appear to improve human NK cell cytotoxicity (312–316). Similarly, CTLA-4 expressed by NK cells inhibited IFN-γ production in response to B7-1 induced by dendritic cells (299). In mice, Kit^+^ CD11b^−^ NK cells capable of B7-H1-dependent immuno-ablative functions were elicited by IL-18 produced by tumor cells. Kit^+^ CD11b^−^ NK cells also reported upregulation of CTLA-4; however, its involvement in tumor progression in NK cell-controlled cancers has not been investigated (300). These studies reveal an undeniable role for this pathway in NK cell-mediated toxicity. However, co-stimulation mediated by CD80-CD28/CTLA-4 being absent in human NK cells has also been proposed (317). Co-stimulation of CD28/B7 was also denied to play any significant part in peripheral NK cells in murine CMV infection (317).

**Figure 4:**
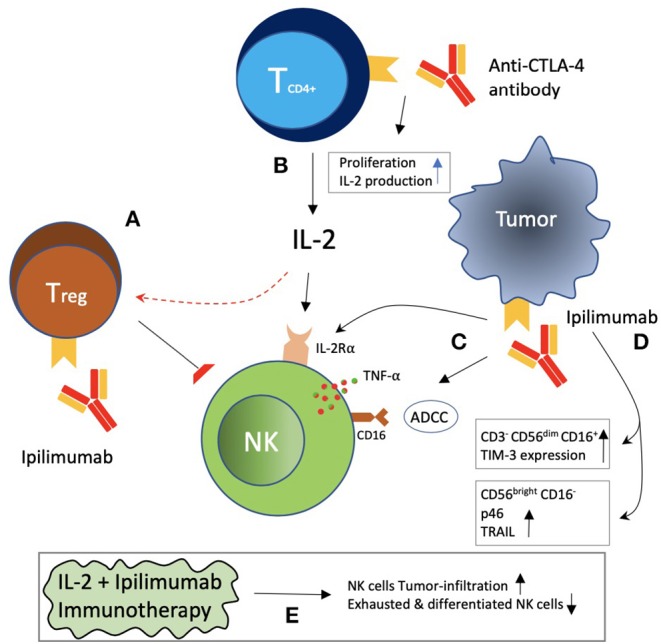
Indirect effects of CTLA-4 blockade on NK cells. **(A)** Treg increase was correlated with NK cell suppression and decrease IL-2 availability to NK cells, which may be reversed with CTLA-4 blockade. **(B)** CTLA-4 blockade with antibodies increased CD4^+^ T-cell proliferation and IL-2 production. **(C)** Ipilimumab blockade of CTLA-4 on tumor cells has been reported to be associated with ADCC, TNF-α release by NK cells, and induction of the IL-2Rα chain on NK cells. **(D)** Ipilimumab was associated with an increased frequency of CD3^−^ CD56^dim^ CD16^+^ NK cells with increased TIM-3 expression. CD56^bright^ CD16^−^ NK cells with increased expression of p46 receptor and TRAIL have also been reported. **(E)** A combination of IL-2 plus ipilimumab was reported to be associated with increased NK cell infiltration of tumor, as well as a decrease in exhausted and differentiated NK cells.

CTLA-4^+^ tumor-infiltrating NK cells embody an anti-CTLA-4 monoclonal antibody-based prospective immunotherapeutic target. Blocking CTLA-4 might relieve the suppressed NK cells in an indirect manner, as shown in Figure 4. The expression of CTLA-4 on Tregs is thought to be essential for their suppressive functions (318). NK cell cytotoxicity-suppression with an increase in CTLA-4 positive Tregs was correlated with poor prognosis in cetuximab-treated head and neck cancer patients (319). Ipilimumab, an anti-CTLA-4 monoclonal antibody, leading to depletion of regulatory T cells, resulted in clinical efficacy in melanoma patients in an Fc-mediated manner, which may be partly due to relieved NK cell cytotoxicity suppressed by Tregs (320, 321). The availability of IL-2 released by activated CD4-positive T cells was also restricted by Tregs, which is involved in NK cell proliferation, accumulation, and activation (322, 323). It has been demonstrated that CTLA-4 engagement on CD4^+^ T cells inhibits IL-2 production and CTLA-4 blockade, leading to a rise in IL-2-producing effector cells, again showing anti-CTLA-4 blockade affecting NK cell cytotoxicity in an indirect way (324, 325). Increased frequency of a subset of NK cells (mature circulating CD3^−^ CD56^dim^ CD16^+^ NK cells) with increased TIM-3 expression had also shown a correlation with clinical outcome in melanoma patients during treatment with anti-CTLA-4 blockade (326). A different subset of NK-cells, CD56^bright^CD16^dim^, was involved in mediating the regression of pancreatic cancer from ipilimumab (327). These cells reported an upregulation of p46-activating receptors and TRAIL. Ipilimumab was also able to trigger ADCC via the engagement of FcyRIIIA receptors present on primary NK cells as well as NK cells and γδT cells activated by IL-2 by reacting with CTLA-4 present on melanoma cell lines and tissues. Furthermore, Ipilimumab and CTLA-4-positive melanoma cell interaction also led to TNF-α release by NK cells (328). Delay in tumor growth and survival prolongation were witnessed in melanoma with a combination of CTLA-4 blockade and IL-2 immunotherapy, indicating synergism. Tumor infiltration of the immune cells, including CD8-positive T cells and NK cells, was increased with CTLA-4 blockade, while a reduction in the proportion of tumor-infiltrating NK cells that were exhausted and differentiated was observed with IL-2 (329). Ipilimumab demonstrated induction of IL-2R α-chain expression on the phenotype of NK cells, with subsequent enhanced response to IL-2 stimulation and cytotoxicity, and this was associated with better clinical response in advanced melanoma patients (330).

## PD-1

Programmed cell Death-1 (PD-1) is expressed on various immune cells, including T (CD4^+^ & CD8^+^), B cells and myeloid cells, NK cells, NKT cells and other innate lymphoid cells (IL2) (298, 306, 331–334). Ligands for PD-1, PD-L1, and PD-L2 upregulation have been reported on in various cancers, and their interactions have led to T-cell inhibition, resulting in immune escape (298, 306). High PD-1 expression on NK cells is detectable in the peripheral blood of approximately a quarter of healthy individuals (335). However, PD-1 expression on NK cells is upregulated in cancer patients such as in ascites of ovarian-carcinoma patients, peripheral blood of Kaposi sarcoma patients, and renal cell carcinoma and multiple myeloma patients (336–340). It is also upregulated on peripheral and tumor-infiltrating NK cells in digestive cancers such as esophageal, gastric, biliary, liver, and colorectal cancer (341). Chronic infections such as HIV (Human Immunodeficiency Virus), HCV (hepatitis C virus), HCMV (Human cytomegalovirus), and *M. tuberculosis* have also shown enhanced PD-1 expression on NK cells (342, 343).

PD-1 expression on NK cells is diverse, varying from cancer to cancer. PD-1 expression is generally lacking in CD56^bright^ NK cells. CD56^dim^ NK cells, however, have demonstrated PD-1 expression that is restricted to the NKG2A^−^ KIR^+^CD57^+^ phenotype, a fully mature NK cell (335). The NKG2A^−^ KIR^+^CD57^+^ phenotype NK cell is considered to have strikingly downregulated activating receptors such as NKp30 and NKp46. Furthermore, a correlation was witnessed between PD-1 expression and NK cell anti-tumor activity impairment, while disrupting PD-1 and PD-L1 interaction by antibody led to partial restoration (344). PTLD pediatric transplant patients have also demonstrated NK cell functional alteration, with increased PD-1 and decreased NKp46 and NKG2D expression (345). On the other hand, CD56^bright^ NK cells were shown to express PD-1 in chronic HCV patients (332). Meanwhile, in patients with digestive cancers, both types of NK cells (CD56^bright^ and CD56^dim^ NK cells) have demonstrated increased PD-1 expression (341). Furthermore, newly identified hepatic resident infiltrated CD3^−^CD49a^+^CD56^+^NK cells in HCC tissues also revealed enormous expression of PD-1 on their surfaces, which was associated with reduced survival in hepatocellular carcinoma patients (341).

NK cell upregulation of PD-1 expression in several cancers suggests a dysfunctional state of the NK cell, probably due to over-stimulation by tumor cells deficient in MHC-I. Comparisons of PD-1^+^ NK cells and PD-1^−^ NK cells have revealed PD-1^+^NK cells to be functionally exhausted, with impaired cytotoxicity and cytokine production and reduced proliferative capability (335, 341). Blockade with anti-PD-1 mAbs has been shown to revert NK cell functional capability. Mouse tumor-resident NK cells exhibit PD-1 expression, and antitumor immune response by NK cells was elicited with anti-PD-1 blockade (346). Antibodies against PD-1 were able to reverse impaired degranulation toward an ovarian carcinoma cell line (335). *In vitro*, NK cell-mediated cytotoxicity toward autologous MM cells was enhanced by anti-PD-1 antibody (339). Killing of mouse glioma stem cells (GL261GSCs) by mouse NK cells was also promoted by PD-1 blockade (347).

In PTLD patients, IFN-γ release, but not cytotoxicity, was enhanced by anti-PD-1 pathway disruption, indicating a partial dependence on the PD-1 pathway (345). Hence, more studies are required to clearly determine the role of PD-1 blockade in the context of NK cells. Blocking PD-L1 has also been shown to improve NK cell-based anti-tumor responses (348). In HN cancer patients, activated PD-1^+^ NK cell dysfunction was mediated by PD-L1 (349). CD56^dim^ PD^+^NK cells expressed in Hodgkin lymphoma were efficiently suppressed by PD-L1-expressing myeloid cells, which was reversed by anti-PD-1 blockade *in vitro* (350). ADCC toward multiple types of carcinoma cells obtained by avelumab, an anti-PD-L1 antibody, was augmented with epigenetic priming of NK cells and tumor (351). In another study, Avelumab triggered NK cells to produce cytokines and mediate the killing of triple-negative breast cancer cells (352). PD-L1-independent killing of CRC cells that were grown in 3D cultures by densely activated primary human NK cells has also been demonstrated (353). Again, it seems that the biology of NK cells in the context of checkpoint inhibition is very complex and needs further exploration.

## B7-H3

B7 homolog 3 protein (B7-H3) is a ligand molecule belonging to the B7-CD28 family for which the receptor, probably present on T cells and NK cells, as it appears to inhibit both T- and NK-cell functions, is yet to be discovered (297). B7-H3 is believed to be both co-stimulatory and co-inhibitory in regulating T-cell functions (354–356). B7-H3 co-stimulates T-cell activation by binding to TLT-2, whereas binding to unidentified receptor(s) leads to co-inhibition of T cells. Meanwhile, it is an inhibitor for NK cells and osteoblastic cells by ligating unknown receptor(s) (355).

There is a limited expression of B7-H3 on various normal tissues such as pancreas, liver, small intestine, colon, heart, thymus, spleen, placenta, and testis (357). However, aberrant expression of B7-H3 is seen in various malignancies in which it is associated with poor outcome, including RCC, breast cancer, lung cancer, esophageal squamous cancer, gastric cancer, pancreatic cancer, gallbladder cancer, colorectal cancer, prostate cancer, ovarian cancer, cervical cancer, endometrial cancer, osteosarcoma, and neuroblastoma (358, 359). Circulating serum B7-H3 levels are significantly higher in patients with lung cancer, renal cell carcinoma, hepatocellular carcinoma, colorectal carcinoma, and glioma than in healthy volunteers (360). Suppression of NK cell-mediated cytotoxicity is one of the various mechanisms involved in tumor evasion by B7-H3-expressing cells (297). Glioma malignancy grade and reduced survival were correlated with the expression of B7-H3 in tumor and endothelial cells. Soluble B7-H3 in the supernatant of glioma cells and cell-bound B7-H3 were able to suppress natural killer cell-mediated tumor cell lysis. Susceptibility to killing was confirmed in an *in vivo* model of B7-H3-silenced glioma cell lines (361). Monoclonal antibody-mediated masking of the 4Ig-B7-H3 molecule, identified as a neuroblastoma-associated molecule, on cell transfectants or on freshly isolated neuroblastoma cells protected it from killing by NK cells (362). Similarly, in a case of neuroblastoma arising in an ovarian cystic teratoma, B7-H3 was expressed in addition to substantial amounts of HLA class-I molecules, suggesting a protective immune-evasive mechanism by neuroblastoma cells against NK cell-mediated lysis. Additionally, receptors such as DNAM-1 (CD226) and CD16 were expressed with lower intensity on NK cells isolated from peritoneal fluid of these patients in comparison to NK cells from the peripheral blood (363). Bispecific killer cell engager (BiKE) treatment, one of the two forms of antibody therapeutics, was demonstrated to significantly inhibit cell growth by inducing natural killer cells when exploring B7-H3 as a potential target in NSCLC (364).

B7-H3 binding Fc-optimized humanized IgG1 mAb, Enoblituzumab, is currently being explored (Table 1). In order to improve functional effectivity, including ADCC, enoblituzumab is Fc-engineered. It has been shown to inhibit tumor growth in renal and bladder carcinoma xenografts positive for B7-H3 (365). MGA271, an Fc-optimized humanized mAb targeting B7-H3, has shown safety and antitumor efficacy in several tumor types that were refractory B7-H3-expressing cancers or where the vasculature of the patients with these cancers were positive for B7-H3, as revealed from an interim analysis of an ongoing dose-escalating phase I study (366). This anti-tumor activity was attributed to an increase in the T-cell repertoire clonality in patients. Further characterization of enoblituzumab including its pharmacological kinetics and dynamics as well as its safety profile, dosage toleration, and antitumor activity against relapsed or refractory solid malignancies with positive expression of B7-H3 receptors in younger patients (children and young adults), is being evaluated in an open-label phase I study (NCT02982941) (367). Orlotamab (MGD009, a humanized B7-H3 x CD3 DART® protein), a bispecific antibody targeting CD3 in addition to B7-H3, developed by MacrGenics is under clinical investigation (NCT03406949) for its safety and efficacy in combination with anti-PD-1 antibody (MGA012) in relapsed or refractory tumors with B7-H3 expression (Table 1). Plenty remains to be unraveled regarding B7-H3, particularly the discovery of its receptors on NK cells and T cells. All in all, B7-H3 presents itself as a potential candidate for checkpoint-based immunotherapy against T cells as well as NK cells.

## Conclusions

Natural killer cells represent a distinct group of anti-tumor response agents with functions like MHC-unrestricted cytotoxicity, cytokine production, and immunologic memory, making them a key player in the innate as well as the adaptive immune response system. The development of several cancers has been associated with the presence of dysfunctional NK cells. Hence, restoration of such NK cells could be a potential option for anti-tumor immunotherapy. One approach to such restoration is the inhibition of immune checkpoints, which is the manipulation of inhibitory receptors on the immune cell surface by cancer cells for immune escape. Immune checkpoint inhibition has been successful in the context of T cells. NK cells have recently been focused on for the same purpose. The immune checkpoint inhibitors, such as monalizumab and lirilumab, aimed at these inhibitory receptors present at the surface of NK cells have been assessed as monotherapy and have shown good safety profiles but mild success in terms of prolonging progression-free survival. Therefore, the combinations of immune checkpoint inhibitors, such as CTLA-4 and PD-1 inhibitors, that are being tried for synergistic response targeting T cells could also be tried in the context of NK cells (368), as anti-PD-1 and anti-PD-L1 inhibitors have also been shown to enhance NK cell-mediated cytotoxicity (346, 348, 352). Similarly, NKG2A potentiating CD8 T-cell immunity induced by cancer vaccines also stresses the potential of combination therapy (141). Hence, a combination of the two, an anti-PD-1 or anti-PD-L1 inhibitor and an NK cell-specific checkpoint inhibitor such as anti-KIR or anti-NKG2A inhibitor, could be of value for combined checkpoint inhibition-based immunotherapy. With the addition of newer checkpoints such as B7-H3, CD200R, CD47, and Siglecs 7/9, it seems more logical to combine these checkpoints for synergistic anti-tumor response.

Another important aspect in the context of immune checkpoint inhibition is the unraveling of the importance of cancer profiling based on biomarkers and the identification of NK cell phenotypes for individual patients in order to get a good idea of where an individual is more likely to get benefit from a given immune checkpoint inhibitor or a combination of inhibitors and cytokine therapy or virotherapy. Inhibitory NK cell receptors may function in different subsets of NK cells or in distinct states of differentiation, such as the acquisition of KIR and loss of NKG2A observed during NK-cell differentiation. Hence, lirilumab will not be effective if the KIR-negative NK cells are responsible for tumor growth (369). Similarly, CD56^bright^ NK cells exhibit potent antitumor responses following IL-15 priming (370). Interleukin-7 also selectively enhances natural kill cytotoxicity mediated by the CD56^bright^ natural killer subpopulation (371). Hence, cytokine therapy would work well if the CD56^bright^ NK cells are required for cancer elimination. Furthermore, as the contribution of several other factors in addition to inhibitory receptors leads to an immune escape of NK cells, for example, downregulation of activating receptors, molecular checkpoints of the activation pathways such as Cbl, GSK-3β, DGKζ, and CIS, and tumor environment suppressors, a combination of strategies countering each of these processes could lead to a more complementary and synergistic potent immune response (369). This would certainly lead to a more comprehensive cancer treatment approach composed of immune checkpoint blockade, augmentation of activating receptors or their molecular checkpoints, and cancer environment checkpoint blockade. Yet another way to improve checkpoint blockade efficacy is its integration with other standard cancer therapeutics, such as surgery, chemotherapy, and radiotherapy, in particular (372, 373). These are some of the areas that should be evaluated in order to increase the clinical efficacy of immune checkpoint blockade in the future.

## Author Contributions

All authors listed have made a substantial, direct and intellectual contribution to the work, and approved it for publication.

### Conflict of Interest

The authors declare that the research was conducted in the absence of any commercial or financial relationships that could be construed as a potential conflict of interest.
